# Parallel evolution of a splicing program controlling neuronal excitability in flies and mammals

**DOI:** 10.1126/sciadv.abk0445

**Published:** 2022-01-28

**Authors:** Antonio Torres-Méndez, Sinziana Pop, Sophie Bonnal, Isabel Almudi, Alida Avola, Ruairí J. V. Roberts, Chiara Paolantoni, Ana Alcaina-Caro, Ane Martín-Anduaga, Irmgard U. Haussmann, Violeta Morin, Fernando Casares, Matthias Soller, Sebastian Kadener, Jean-Yves Roignant, Lucia Prieto-Godino, Manuel Irimia

**Affiliations:** 1 Centre for Genomic Regulation, Barcelona Institute of Science and Technology (BIST), Barcelona 08003, Spain.; 2 Francis Crick Institute, London, UK.; 3 Centro Andaluz de Biología del Desarrollo (CABD), CSIC-Universidad Pablo de Olavide-Junta de Andalucía, Seville, Spain.; 4 Department of Genetics, Microbiology and Statistics and Institut de Recerca de la Biodiversitat (IRBio), Universitat de Barcelona, Barcelona, Spain.; 5 Center for Integrative Genomics, Génopode Building, Faculty of Biology and Medicine, University of Lausanne, CH-1015 Lausanne, Switzerland.; 6 Biology Department, Brandeis University, Waltham, MA, USA.; 7 Department of Life Science, School of Health Sciences, Birmingham City University, Birmingham B5 3TN, UK.; 8 Institute of Molecular Biology (IMB), Mainz, Germany.; 9 School of Biosciences, College of Life and Environmental Sciences, University of Birmingham, Edgbaston, Birmingham B15 2TT, UK.; 10 Birmingham Centre for Genome Biology, University of Birmingham, Edgbaston, Birmingham B15 2TT, UK.; 11 Institute of Pharmaceutical and Biomedical Sciences, Johannes Gutenberg-University Mainz, Staudingerweg 5, 55128 Mainz, Germany.; 12 Universitat Pompeu Fabra (UPF), Barcelona 08003, Spain.; 13 ICREA, Barcelona, Spain.

## Abstract

Alternative splicing increases neuronal transcriptomic complexity throughout animal phylogeny. To delve into the mechanisms controlling the assembly and evolution of this regulatory layer, we characterized the neuronal microexon program in *Drosophila* and compared it with that of mammals. In nonvertebrate bilaterians, this splicing program is restricted to neurons by the posttranscriptional processing of the *enhancer of microexons* (eMIC) domain in *Srrm234*. In *Drosophila*, this processing is dependent on regulation by Elav/Fne. eMIC deficiency or misexpression leads to widespread neurological alterations largely emerging from impaired neuronal activity, as revealed by a combination of neuronal imaging experiments and cell type–specific rescues. These defects are associated with the genome-wide skipping of short neural exons, which are strongly enriched in ion channels. We found no overlap of eMIC-regulated exons between flies and mice, illustrating how ancient posttranscriptional programs can evolve independently in different phyla to affect distinct cellular modules while maintaining cell-type specificity.

## Teaser

Neuronal microexons in flies and mammals affect disparate cellular modules while sharing regulatory and cell-type specificity.

## INTRODUCTION

Protein-coding genes in metazoans undergo multiple mRNA processing steps before they are ready for translation. One pivotal step is the removal of introns, mediated by the interaction of the splicing machinery and other related proteins with the pre-mRNA. Splice site selection is not deterministic, and several mRNA products can be produced from the same gene in a process known as alternative splicing (AS). AS can greatly expand the coding capacity of metazoan genomes, with notable examples including the *Down syndrome adhesion molecule 1* (Dscam1) from *Drosophila melanogaster*, which can generate more than 35,000 alternatively spliced isoforms from a single gene (*1*).

In evolutionary terms, AS can serve similar functions as gene duplication since it allows for the exploration of new coding capabilities without affecting preexisting gene functionality (*2*, *3*). In metazoans, neural tissues have particularly exploited the potential brought by AS and present the highest number of tissue-enriched exons (*4*, *5*). These neurally enriched isoforms have been implicated in key aspects of neuronal biology including neurogenesis, axon guidance and growth, synapse formation, and synaptic plasticity (*6*, *7*). Neural splicing programs are coordinated by the action of RNA binding proteins (RBPs) that are predominantly expressed in this tissue and can modulate hundreds of splicing decisions genome-wide (*8*). The importance of these splicing choices in the brain is underscored by the widespread association between splicing alterations and neurological disorders such as in autism spectrum disorder, spinal muscular atrophy, amyotrophic lateral sclerosis, Huntington’s disease, or intellectual disability, among others (*9*, *10*).

Among these programs, transcriptomic analyses across vertebrate tissues and human brain samples uncovered a highly conserved set of very short neural-enriched exons: microexons. These are down-regulated in some autistic patients (*11*), and their misregulation in mouse models leads to a wide range of neurological phenotypes (*12*–*14*). Splicing of neural microexons is regulated by the combinatorial action of several splicing factors. The serine/arginine repetitive matrix 4 protein SRRM4 and its paralog SRRM3 are the master regulators of microexon splicing, being sufficient to promote inclusion of ~90% of neural microexons when ectopically expressed in non-neural cells (*11*, *15*). Many neural microexons are also repressed by PTBP1 (polypyrimidine tract binding protein 1) in non-neural samples (*16*, *17*), thereby reinforcing their switch-like profile across tissues. A recent high-throughput study searching for microexon regulators identified two additional factors, RNPS1 (RNA binding protein with serine rich domain 1) and SRSF11 (serine and arginine rich splicing factor 11), which cooperate with SRRM4 to assemble an exon definition complex that facilitates microexon splicing (*18*). Expanding microexon profiling beyond vertebrates revealed that neural microexons originated in bilaterian ancestors in association with the appearance of a novel domain in the ancestral *Srrm234* gene that is necessary and sufficient for neural microexon splicing: the *enhancer of microexons* or “eMIC” domain (*15*). The eMIC domain is a specialized type of Arg/Ser-rich domain that interacts with the earliest factors involved in exon recognition, U2-auxiliary factors 1 and 2 (U2AF1/2) and splicing factor 1 (SF1), thereby promoting spliceosome assembly on neural microexons (*15*). Neural expression of the eMIC domain is regulated transcriptionally through the expression of *Srrm3* and *Srrm4* in vertebrates, both containing the eMIC domain but through posttranscriptional processing of *Srrm234* in nonvertebrates (*15*).

Here, we address the regulation, functional impact, and evolution of the neural microexon program in a nonvertebrate. For this, we generated *D. melanogaster* flies with eMIC loss of function, as well as transgenic lines for cell type–specific expression of different variants of the *Srrm234* gene. eMIC-null flies display an array of neurological defects, including alterations in locomotion, aging, sleep, metabolism, and bang sensitivity. Expression of transgenic *Srrm234* variants in different cell types underscores the relevance of spatially and quantitatively regulating eMIC activity in *Drosophila*. Lack of eMIC activity results in genome-wide down-regulation of short alternative exons, affecting up to one-third of all neural exons in *D. melanogaster*. By profiling AS in over 700 RNA sequencing (RNA-seq) samples, we generated a catalog of all tissue- and cell type–specific exons, with a focus on eMIC-dependent exons. We also characterized the cis-regulatory code associated with eMIC-dependent splicing in *Drosophila*, highlighting differences and similarities with mammals. Notably, despite the remarkable cell type–specific conservation, we only found four exons in equivalent positions between the fly and mammalian eMIC splicing programs, indicating that both programs evolved largely independently from an ancestral neuronal-specific program.

## RESULTS

### Regulated 3′ end processing of *Srrm234* ensures strict eMIC neural expression

**Fig. 1.**
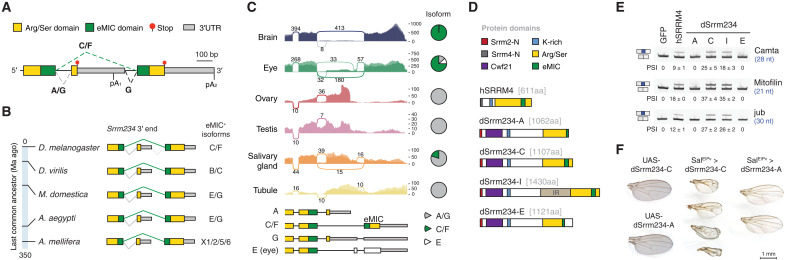
Posttranscriptional regulation of eMIC domain expression in *D. melanogaster*. (**A**) Genomic region encompassing the 3′-most terminal exons of the *Srrm234* (CG7971) gene and the corresponding protein domains encoded therein. Arg/Ser, arginine/serine rich; 3′UTR, 3′ untranslated region; bp, base pairs. Putative AS and polyadenylation (pA) sites are indicated together with their associated reference transcripts (A, C, F, and G). (**B**) Genomic architecture of the 3′ end of the *Srrm234* locus in different insect species. Reference isoforms encoding the eMIC domain are indicated. Ma ago, million years ago. (**C**) Sashimi plots of RNA-seq data from different tissues at the *Srrm234* 3′ end region. Average numbers of reads spanning each splice junction between male and female samples are indicated. *Y* axis represents absolute number of mapping reads (without normalization for library size). Bottom: Main transcript isoforms annotated for the *Srrm234* gene (FlyBase annotation). Pie charts depict isoform usage quantified on the basis of junction reads only. Data from FlyAtlas 2 (*19*). (**D**) Protein domains of *Drosophila* (d) Srrm234 isoforms and human (h) SRRM4. IR, intron retention; aa, amino acids; K, lysine; Srrm2/4-N, conserved regions at Srrm2/4 N termini. (**E**) Reverse transcription polymerase chain reaction (RT-PCR) assays for alternatively spliced exons in SL2 cells overexpressing different Srrm234 isoforms. PSI, percentage spliced in; nt, nucleotides. Numbers indicate mean and SD from three replicates. (**F**) Representative pictures of fly wings overexpressing either dSrrm234-C or dSrrm234-A under the control of *spalt* (Sal^E|PV^-GAL4), active in the center of the wing blade (*20*), and their respective controls.

The *Srrm234* gene in *Drosophila* can produce disparate protein isoforms based on the posttranscriptional processing at its 3′ end (Fig. 1A and fig. S1). Alternative last exon selection at this locus depends on a combination of AS and alternative polyadenylation (APA) events (Fig. 1A). The proximal non–eMIC-encoding exon can be expressed either as a terminal exon making use of its own polyadenylation (poly-A) site (pA_1_ site in Fig. 1A, isoform A) or as a “poison” exon for the eMIC-expressing isoform when the distal poly-A site is used (pA_2_ site in Fig. 1A, isoform G). Translation of the distal exon encoding the eMIC domain requires both distal poly-A usage and skipping of the proximal exon (C and F isoforms). This particular genetic architecture encoding the eMIC domain as an alternative last exon of the *Srrm234* gene is conserved at least within holometabolous insects (Fig. 1B).

We analyzed isoform usage at this region using tissue-specific RNA-seq data from FlyAtlas 2 (*19*) and found that eMIC expression (isoforms C/F) is strongly biased toward neural tissues (brain and eyes), whereas other tissues mainly express *Srrm234* isoforms with no eMIC (A/G) (Fig. 1C and fig. S1). This analysis also revealed a fourth splice variant that is only expressed in the eye (Fig. 1C). We cloned representative *Srrm234* isoforms to test their splicing activity on neural microexons by heterologous expression in *Drosophila* SL2 cells (Fig. 1, D and E). We chose four isoforms for this experiment: the two reference isoforms A and C, which only differ at the C terminus where the full eMIC is encoded in isoform C but not in A; a variant of isoform C containing a protein-coding neural-retained intron as in reference isoform F, which we termed isoform I (see Materials and Methods); and the newly identified eye-specific isoform, which we named isoform E. As a control, we used the human ortholog SRRM4 (hSRRM4), which we have previously shown to promote inclusion of short endogenous neural exons in this system (*15*). Consistent with previous studies, only the proteins harboring the complete eMIC domain were able to promote inclusion of short neural exons (Fig. 1E and fig. S2A).

To investigate the functional relevance of the restriction of the eMIC to neural tissues, we ectopically expressed the eMIC domain in a non-neural tissue (the wing) by generating transgenic flies with different *Srrm234* isoforms under the control of the GAL4-specific UAS enhancer (Fig. 1F and fig. S2, B and C). Expression of isoform C, but not A, in the entire wing pouch under a nubbin (*nub*) driver line promoted the inclusion of short neural exons, preventing the formation of adult wings and severely affecting haltere morphology (fig. S2, B and C). Expression in the center of the wing blade only, under a *spalt* (*Sal^E|PV^*) driver (*20*), generated bubbles, shortening, and blister phenotypes (Fig. 1F). These results highlight the detrimental effects of eMIC expression outside neuronal tissues, and the latter may indicate additional non–cell autonomous effects, as defects spread beyond the delimited area of *Sal^E|PV^* expression. Together, these results show that *Srrm234* last exon selection, and hence eMIC domain expression, needs to be tightly regulated to restrict its activity to the neural system.

### Altered eMIC expression levels result in widespread neurological defects in *Drosophila*

**Fig. 2.**
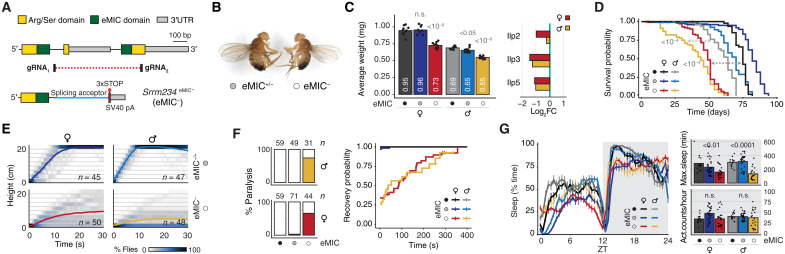
Physiological alterations associated with eMIC domain loss of function. (**A**) CRISPR strategy to generate mutant flies with eMIC-specific deletion at the *Srrm234* locus. Dotted red line spans the deleted genomic region, replaced by an integration cassette (fig. S2D). (**B**) Left: eMIC^+/−^ male 1-day-old fly from the cross with w^1118^. Right: eMIC^−^ male. Photo credit: Antonio Torres-Méndez. (**C**) Left: Average weight of flies less than 24 hours after hatching. White numbers indicate mean values. *P* values from two-sided *t* tests comparing with w^1118^ controls. Right: Log_2_ fold change in expression levels in eMIC^−^ adult brain respect to control brains as quantified from RNA-seq data. (**D**) Longevity assay, *n* = 100 flies per sex and genotype. *P* value from log-rank tests. (**E**) Kymographs displaying *Drosophila* negative geotaxis behavior. Colored lines indicate average height at each time point. (**F**) Sensitivity of adult flies to mechanical stimulation (10-s vortex). Left: Percentage of paralyzing (bang-sensitive) flies per sex. *n*, number of flies tested. Right: Probability of recovering from mechanical-induced paralysis over time. (**G**) Sleep patterns of 3-day-old flies in 12-hour light/12-hour dark cycles. Left: Average time flies spend sleeping (inactive for ≥5 min) at different times of the light/dark cycle. ZT, Zeitgeber time; vertical lines, SEM. Top right: Maximum sleep episode during the night. Bottom right: Total number of activity counts per hour during the night. *P* value from Mann-Whitney *U* tests comparing eMIC^−^ with control flies. All panels: Filled circles correspond to number of eMIC^+^ alleles (black, +/+; gray, +/−; empty, −/−). *n*, number of individual flies tested, n.s., nonsignificant or *P* > 0.05.

To characterize the neural microexon splicing program in *Drosophila*, we generated eMIC-specific knockout flies (*Srrm234*^eMIC-^; hereafter eMIC-) via CRISPR-Cas9 targeting the 3′ region of *Srrm234* with two guide RNAs (gRNAs) and replacing it with an integration cassette (Fig. 2A; figs. S1 and S2, D to F; and Materials and Methods). Only ~15% of eMIC^−^ flies reach pupal stage. Furthermore, surviving eMIC^−^ adult flies are smaller than controls, with a 20% reduction in body weight at hatching, and have reduced life span (Fig. 2, B to D). These size and weight reductions are correlated with reduced levels of neuronally secreted insulin-like peptides: Ilp2, Ilp3, and Ilp5 (*21*), in both adult and larval brains (Fig. 2C and fig. S2G). As expected, reverse transcription polymerase chain reaction (RT-PCR) assays on fly heads showed that eMIC deficiency leads to skipping of short neural-enriched exons (fig. S2H).

To investigate the functional role of the AS program regulated by eMIC activity, we ran a battery of behavioral assays on eMIC-deficient flies. Both male and female eMIC^−^ flies have tremors and defects in self-righting—a complex motor sequence that allows animals to adopt a locomotion position if turned upside down (movie S1). Performance of eMIC^−^ flies in the negative geotaxis assay is very poor in both sexes but more pronounced in males (Fig. 2E). eMIC^−^ flies are also bang sensitive, i.e., they undergo seizures after mechanical stress, with a recovery time similar to classical bang-sensitive mutants (Fig. 2F) (*22*). We monitored daily activity patterns and found that, despite these alterations, overall activity was similar in control and eMIC-null flies (Fig. 2G). However, mutant flies sleep less and have more fragmented sleep, a phenotype that becomes more pronounced in older flies (Fig. 2G and fig. S2I).

**Fig. 3.**
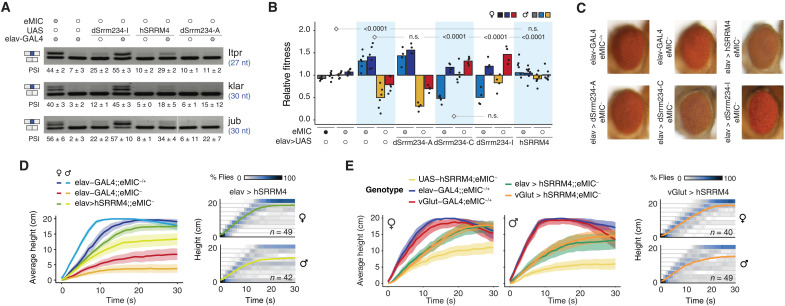
Phenotypic rescue and gain of function in Srrm234 transgenic flies. (**A**) RT-PCR assays for short neural exons from female fly heads. Numbers indicate mean and SD from three replicates. Filled circles indicate the genotype for each allele (black, +/+; gray, +/−; empty, −/−). (**B**) Relative fitness of flies with varying number of endogenous and transgenic eMIC alleles. See fig. S3A for a detailed mating scheme. *P* values from chi-square tests on the observed frequencies of genotypes per cross, compared with crosses marked with a diamond. (**C**) Representative pictures of eyes from young flies expressing Srrm234 isoforms pan-neuronally in the eMIC^−^ background. (**D** and **E**) Kymographs displaying the negative geotaxis behavior of *Drosophila* eMIC^−^ flies upon expression of UAS-*hSRRM4* pan-neuronally using an elav-GAL4 driver line (D and E) or in glutamatergic neurons only, under a vGlut-GAL4 line (E). Colored lines indicate average height at each time point, and ribbons denote 95% nonparametric bootstrap confidence intervals. *n*, number of individual flies tested.

Using the GAL4-UAS system, we performed rescue experiments by expressing either human SRRM4 or *Drosophila* Srrm234 isoforms C/I pan-neuronally in the eMIC^−^ background. As a negative control, we expressed dSrrm234 isoform A, which lacks a functional eMIC domain. We first calculated the relative fitness of each genotype, defined as the proportion of emerging adults of that genotype over the expected Mendelian proportions (fig. S3A). eMIC^−^ flies expressing dSrrm234-A in neurons showed the same low fitness of eMIC^−^ flies relative to their eMIC^−/+^ controls, demonstrating that the A isoform lacking microexon regulatory activity cannot rescue eMIC mutant phenotypes (Fig. 3, A and B, and fig. S3, A and B). In contrast, both dSrrm234-C and dSrrm234-I can restore the inclusion of neural microexons and equally overcome fitness defects in eMIC^−^ flies. However, unexpectedly, the relative fitness of control (eMIC^−/+^) flies overexpressing these protein forms was significantly reduced, indicating a deleterious effect associated with excessive levels of eMIC activity, particularly in males (Fig. 3, A and B, and fig. S3, A and B). Moreover, dSrrm234-C and dSrrm234-I pan-neuronal overexpression also caused several neurological alterations, including rough eye patterning (Fig. 3C), problems with wing expansion and wing and leg positioning (fig. S3C), and severe locomotion defects (movie S2). Nevertheless, functional rescue could be observed for both the sleep and bang sensitivity phenotypes (fig. S3, D and E). *eMIC* expression levels in the pan-neuronal dSrrm234-I rescue heads were not higher than controls, yet some neural short exons had increased inclusion levels (Fig. 3A and fig. S3, B and F). This suggests that the phenotypic alterations observed in the rescue flies may come from abnormal eMIC expression in cells with low endogenous eMIC activity or other potential gain-of-function side effects.

Beyond these, pan-neuronal expression of human SRRM4 leads to intermediate microexon inclusion levels (Fig. 3A and fig. S3B) and shows different phenotypic rescues. Specifically, it restores relative fitness defects of eMIC^−^ flies despite affecting overall viability (Fig. 3B and fig. S3A) and partially rescues the fragmented sleep phenotype but not the bang sensitivity (fig. S3, D and E). Furthermore, these flies show significantly improved performance on negative geotaxis assays compared to eMIC^−^ in both sexes (Fig. 3D). Together, the observed improvements in behavioral assays by restoring microexon inclusion levels seem to rely on a balance of rescue and gain-of-function effects brought by the different Srrm234 isoforms and underscore that eMIC activity needs to be tightly regulated even within neurons for their correct functioning.

### The eMIC AS program regulates neuronal excitability

With the aim of identifying the relative share of network versus cell-autonomous effects on eMIC^−^ neurological alterations, we next rescued microexon inclusion in eMIC^−^ flies by expressing hSRRM4 only in glutamatergic neurons, which include motoneurons and a restricted number of interneurons in the ventral nerve cord (VNC) (*23*), under the regulation of vGlut-GAL4 (*24*). Unexpectedly, rescue in glutamatergic neurons alone restored climbing performance to the same extent as pan-neuronal rescue (Fig. 3E), indicating that this phenotype mainly stems from cell-autonomous eMIC deficiency in this neuronal population.

**Fig. 4.**
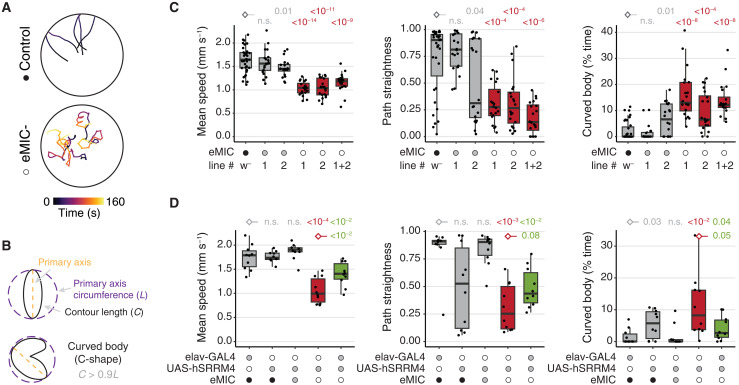
Alterations in locomotor behavior in eMIC^−^ larvae. (**A**) Representative locomotion tracks of free-crawling third-instar larvae (L3). (**B**) Definition of C-shape/curved-body behavior. (**C** and **D**) Quantification of different parameters describing L3 larvae free crawling behavior. In box plots, central lines indicate median values, box limits mark interquartile ranges (IQRs), and whiskers denote 1.5 IQR. *P* values from Welch (speed) or Mann-Whitney *U* tests (path straightness and curved body patterns), from the comparison with samples labeled with a diamond. Filled circles indicate the genotype for each allele (black, +/+; gray, +/−; empty, −/−). Line “1+2” corresponds to the F1 trans-heterozygous from crossing two independent eMIC^−^ lines from the CRISPR-Cas9 genome editing.

**Fig. 5.**
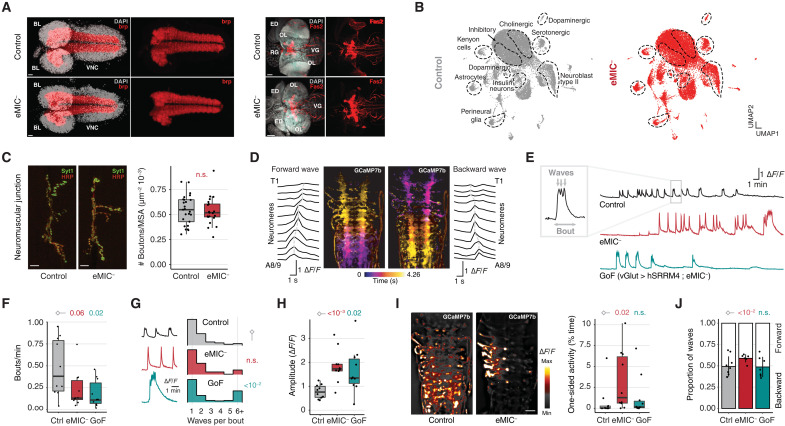
Neuroanatomy and fictive locomotion in eMIC^−^ larvae. (**A**) Confocal images of control and eMIC^−^ larval CNSs using antibodies against bruchpilot (brp; nc82) at L1 stage and fasciclin 2 (Fas2) at L3 stage. BL, brain lobe; OL, optic lobe; VG, ventral ganglia; ED, eye disc; RG, ring gland; DAPI, 4′,6-diamidino-2-phenylindole. Scale bars, 20 and 50 μm in brp and Fas2 stainings, respectively. (**B**) Single-cell RNA-seq data visualized using the UMAP algorithm after integrating control and mutant datasets. Gene markers used for identification of cell populations are included in fig. S4. (**C**) Synaptic bouton quantification at the larval NMJ for control and eMIC^−^ flies based on immunostaining against synaptotagmin (Syt1) and anti–horseradish peroxidase (HRP) to mark neuronal membranes. MSA, muscle surface area. Scale bars, 10 μm. (**D**) Representative activity patterns of motoneurons across VNC segments (neuromeres) during a forward and a backward wave of fictive locomotion, from imaging with the GCaMP7b calcium indicator. T1 and A8-9, first thoracic and last abdominal segments, respectively. (**E**) Representative traces of the mean activity across all segments of the VNC in fictive locomotion experiments for control, eMIC^−^, and motoneuron gain of function, GoF (vGlut > *hSRRM4*; eMIC^−^), larvae. (**F** to **H**) Parameters describing VNC activity patterns during fictive locomotion: number of bouts per minute (F), number of waves per bout (G), and peak amplitude (H). *P* values from Welch (F and H) or Mann-Whitney *U* (G) tests. (**I**) Representative symmetrical and one-sided activity events. Scale bar, 25 μm. Right: Frequency of one-sided events. *P* values from Mann-Whitney *U* tests. (**J**) Proportion of forward and backward waves during fictive locomotion. *P* values from logistic regression. Different panels: F, fluorescence.

To further investigate the mode of action of the eMIC-regulated AS program, we then focused on the *Drosophila* larvae. We first assessed free crawling behavior in third-instar larvae and found that the neurological-associated phenotypes were also present at this stage: eMIC^−^ larvae crawl more slowly, perform more turns with less straight paths, and display unusual unilateral body-wall contractions that lead to C-shape behavior (Fig. 4, A to C). Similar to adult climbing assays, these phenotypes could be partially rescued by pan-neuronal expression of *hSRRM4* (Fig. 4D). Despite the abnormal crawling behavior, examination of overall central nervous system (CNS) morphology revealed no differences between control and eMIC^−^ larvae (Fig. 5A). Moreover, single-cell RNA-seq of eMIC^−^ and control L1 CNS at ~2× coverage (~19,000 cells per genotype) revealed no defects in the generation of major cell types (Fig. 5B), which could be readily identified on the basis of known marker genes (fig. S4). We further examined, in detail, motoneuron axonal terminals and the synapses between motoneurons and muscles at the neuromuscular junction (NMJ) and also found no alterations in eMIC^−^ larvae (Fig. 5C). Together, these results indicate that abnormal crawling behavior is not due to major developmental or morphological alterations.

Therefore, we reasoned that motor phenotypes could be due to defects in neuronal activity. To test this hypothesis, we examined motoneuron activity in the VNC by expressing a genetically encoded calcium indicator (UAS-GCaMP7b) in all glutamatergic neurons (vGlut-GAL4). When isolated, *Drosophila* larval CNS produces spontaneous VNC activity patterns that recapitulate the sequence of muscle activation during locomotion, a process referred to as fictive locomotion (*25*). This occurs in bouts of activity, with each bout made up of several forward and/or backward waves (Fig. 5, D and E, and fig. S5A). One wave represents an increase in GCaMP fluorescence, which travels along the anteroposterior axis of the VNC (Fig. 5D). Neuronal activation correlates with turning and crawling (forward and backward), albeit it is 10 times slower than during actual behavioral sequences (*25*, *26*). We found that the CNSs of eMIC^−^ larvae generated activity bouts at slightly reduced rates compared to controls and that this phenotype could not be rescued by expressing *hSRRM4* in glutamatergic neurons (Fig. 5F). However, whereas control and eMIC^−^ CNSs generated similar number of waves per bout, vGlut *hSRRM4* gain of function partially compensated for the low number of bouts by increasing the number of waves per bouts (Fig. 5G). This indicates that, while the number of bouts might be a property of the whole network, neurons with a functioning eMIC program can cell-autonomously regulate their excitability to generate a higher number of waves. Supporting the dysregulation of neuronal excitability in eMIC^−^ larvae, we found that their calcium waves had significantly higher amplitudes than control (Fig. 5H, fig. S5B, and movie S3). This phenotype was partially, but not completely, rescued by the expression of *hSRRM4* (Fig. 5H). Last, we found that eMIC^−^ CNSs generated a high number of spontaneous unilateral activity events (Fig. 5I), mirroring our behavioral experiments, where eMIC^−^ larvae displayed unusual unilateral body-wall contractions leading to C-shape behavior (Fig. 4B). Moreover, it also generated a higher proportion of backward waves, which could partially explain the lower speed of eMIC^−^ in behavioral assays (Fig. 5J). Notably, these phenotypes could be fully rescued by restoring eMIC expression with *hSRRM4* in glutamatergic neurons (Fig. 5, I and J), mirroring the partial rescue of the corresponding behavioral phenotypes in freely crawling larvae (Fig. 4D). Together, these results point to an important role of the eMIC-regulated AS program in controlling neuronal activity.

### The eMIC domain regulates short neural exons genome-wide in *Drosophila*

**Fig. 6.**
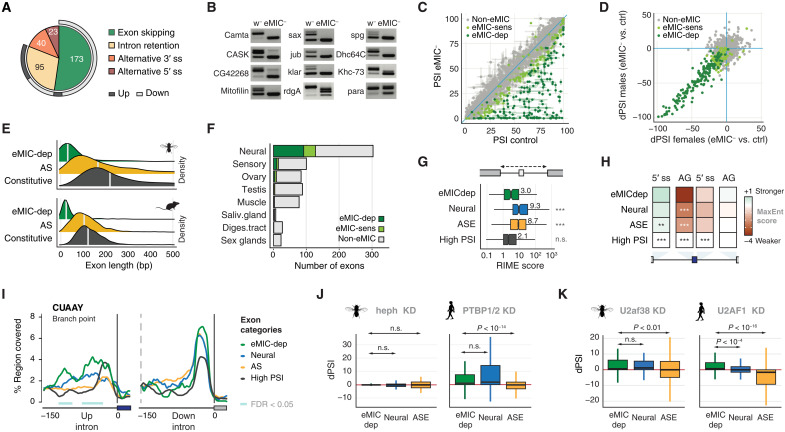
AS regulation by the eMIC domain and its associated cis-regulatory code. (**A**) AS events affected by eMIC insufficiency in adult brains. Up/down, higher/lower inclusion in eMIC^−^ samples. (**B**) RT-PCR validations in control (w^−^) and eMIC^−^ fly heads. (**C**) Genome-wide alterations for alternatively spliced exons in eMIC^−^ adult fly brains. Error bar ends mark the PSI values from male and female samples independently. For exon classification rules, see Materials and Methods. (**D**) Sex comparison for the change in exon inclusion (ΔPSI) between eMIC^−^ and control adult brains. (**E**) Size distribution of eMIC-regulated, AS, and constitutive exons in *Drosophila* and mouse. (**F**) Proportion of tissue-regulated exons (fig. S6E) affected by the knockout of the eMIC domain. (**G**) Ratio of intron to mean exon length (RIME) score for introns harboring different types of cassette exons (table S2). (**H**) Maximum entropy scores for the 5′ splice site and AG region, relative to constitutive (High PSI) exons. (**I**) RNA map for the branch-point sequence (BPS) consensus in *Drosophila* (CUAAY) in introns surrounding different types of exons. Colored rectangles indicate regions with a significant difference in the motif coverage [false discovery rate (FDR) < 0.05] compared to the ASE group. Sliding window, 27 nt. (**J** and **K**) PSI change (dPSI) of different groups of exons upon knockdown (KD) of splicing factors in *Drosophila* SL2 cells and human embryonic kidney (HEK) 293 cells. (J) Knockdown of *heph* (*Drosophila PTBP1/2/3* ortholog) and *PTBP1*/*2*. (K) Knockdown of *U2af38* (*Drosophila U2AF1* ortholog) and *U2AF1*. Data are from (*34*, *36*, *37*, *75*). Different panels: *P* values correspond to the comparison with the eMIC-dependent group (Mann-Whitney *U* tests); *P < 0.01, **P < 0.001, and ***P < 10^−4^.

To comprehensively characterize the splicing program regulated by the eMIC domain in *D. melanogaster*, we sequenced eMIC^−^ and control adult brains and larval CNSs and quantified AS using vast-tools (*11*, *15*). Focusing on AS events within coding sequences, the predominant type of AS affected were cassette exons followed by retained introns (a third of which is associated to the affected exons) (Fig. 6A and fig. S6A). The vast majority of these exons showed increased skipping in the mutant samples (161 of 173 regulated exons; Fig. 6, A to C), highlighting the role of the eMIC as a positive regulator of exon inclusion, as it has been described for Srrm3 and Srrm4 in mammals (fig. S6B) (*12*, *14*, *15*, *17*). Similar results were obtained when using brain samples from FlyAtlas 2 as independent controls, in line with a mutation-specific effect with little-to-none strain specificity (fig. S6C). Next, we classified exons into three groups on the basis of their splicing changes in eMIC^−^ brains: eMIC-dependent (|ΔPSI| ≥ 20, 170 exons), eMIC-sensitive (20 > |ΔPSI| ≥ 10, 128 exons), and non–eMIC-regulated exons (Fig. 6C and Materials and Methods). These exons showed similar inclusion levels between males and females (Fig. 6D), with only 18 eMIC-dependent exons having some mild sex differences in either control or eMIC^−^ adult brains (fig. S6D). Similar to results from mouse Srrm4 targets (*15*), *Drosophila* eMIC targets are much shorter than other alternative exons and constitutive exons (Fig. 6E), corroborating its ancestral role in regulating the inclusion of microexons.

To place the eMIC splicing program within the broader AS landscape of *Drosophila*, we analyzed published transcriptomic datasets (table S1) (*19*, *27*, *28*) using vast-tools and searched for alternatively spliced exons with strong tissue-level regulation. Similar to previous reports (*5*, *29*), we found that neural samples showed the highest prevalence of both tissue-enriched and tissue-depleted exons (fig. S6E). Sensory organs (eye and antenna) displayed a splicing signature that was similar to other neural tissues but with dozens of additional specifically enriched exons, particularly in the eye (fig. S6E). We found that up to one-third of all neural-enriched exons genome-wide are regulated by the eMIC domain (92 eMIC-dep. and 36 eMIC-sens. exons of 303 neural-enriched exons with sufficient read coverage in our samples; Fig. 6F and fig. S6F), qualifying it as a master regulator of neural-specific splicing in *Drosophila*. In addition, AS of the vast majority of these exons was predicted to generate alternative protein isoforms (fig. S6G), suggesting a prominent role fine-tuning the neuronal proteome. Last, we also found numerous muscle-enriched exons, in addition to the previously described splicing singularity of the gonads and sex glands (fig. S6E) (*5*, *29*), which were largely not regulated by the eMIC domain (Fig. 6F). To facilitate AS research in *Drosophila*, we made these AS profiles publicly available at the VastDB website (*30*) (vastdb.crg.eu; example in fig. S6H).

### eMIC-dependent exons display a unique cis-regulatory code

To decipher the regulatory logic of eMIC-dependent splicing, we profiled their sequence features and compared them to other types of exons, including neural non–eMIC-regulated, other alternatively spliced exons (ASEs), cryptic, and constitutive exons (Fig. 6, G to I; fig. S7; and table S2). As shown above, eMIC-dependent exons have a median length of 31 nucleotides (nt), notably shorter than all other exon types in the *Drosophila* genome (fig. S7A). This short exon size is accompanied by short surrounding introns, closer in size to those neighboring constitutive exons rather than other types of alternatively spliced exons (fig. S7A). These very short exon and intron lengths result in low ratio of intron to mean exon length (RIME) scores, usually associated with splicing by intron definition (*31*), very similar to the regime of constitutively spliced introns in *Drosophila* and unlike most other alternatively spliced exons (Fig. 6G) (*32*). eMIC-dependent exons are further characterized by extremely weak 3′ splice site (ss) regions and are also associated with weak 5′ ss but strong upstream 5′ ss compared to constitutive exons (Fig. 6H), unlike mammalian microexons (*11*, *15*). In addition, we found a very unique motif architecture in the 3′ ss region: eMIC-exons have long AG exclusion zones, enrichment for UGC motifs close to the 3′ ss, longer polypyrimidine tracts enriched for alternating UCUC motifs, and strong branch-point sequences (BPSs; CUAAY motif) (Fig. 6I and fig. S7B). The downstream BPS is unusually strong, especially when compared to constitutive exons (Fig. 6I). Apart from the latter and the weak 5′ ss, all other features are also observed in mammalian eMIC targets (fig. S7D) (*15*, *17*). This cis-regulatory architecture is largely unique to eMIC-dependent regulation and not a general property of short exons (fig. S7, D to H).

We hypothesized that the strong definition of the upstream and downstream splice sites, together with the very short length of the surrounding introns, may facilitate skipping of eMIC-exons outside the neural system, rendering specific repressive trans-acting factors unnecessary in *D. melanogaster*. To test this hypothesis, we studied the role in *Drosophila* of the main repressor of Srrm4-dependent exons in mammals (Ptbp1) (*16*, *17*). We analyzed two RNA-seq datasets upon knockdown (KD) of *Hephaestus* (*heph*), the *D. melanogaster Ptbp1/2/3* ortholog, in fly embryos (*33*) and SL2 cells (*34*), and found no evidence for a role in repressing the inclusion of eMIC targets, unlike for equivalent experiments in mammalian cells (Fig. 6J and fig. S8A) (*16*, *17*). To identify potential repressors in an unbiased manner, we also analyzed RNA-seq data from KD experiments from the modENCODE atlas and others (*34*–*36*) for dozens of RBPs in SL2 cells (which endogenously do not express eMIC-dependent exons). This revealed very few factors that might mediate exon repression specifically for eMIC-dependent exons (fig. S8, B and C). Unexpectedly, the top candidate from this analysis was *U2af38* (fig. S8C), the *D. melanogaster* ortholog of mammalian U2-snRNP auxiliary factor 1, *U2af1*, involved in the recognition of the AG dinucleotide at the 3′ ss, and an interacting partner of the eMIC domain (*15*). This repressive role of *U2af38* was common to other neural exons, but not other alternatively spliced exons (Fig. 6K and fig. S8C). A similar negative effect on SRRM4-regulated exons was also observed upon KD of *U2AF1* in human embryonic kidney (HEK) 293 cells (Fig. 6K), and cis-regulatory features of eMIC-dependent and U2AF1-repressed exons are notably similar (*37*), suggesting a previously overlooked conserved mechanism across bilaterians.

### Integration of the eMIC splicing program with other regulatory networks

**Fig. 7.**
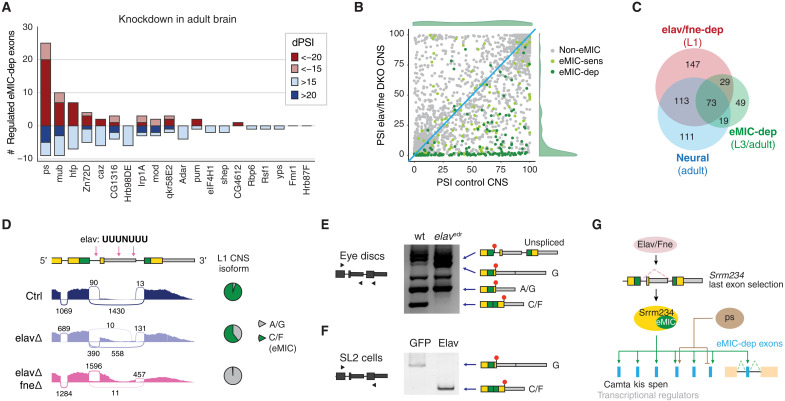
Integration of the eMIC splicing network with other neuronal regulatory programs. (**A**) Cross-regulation of eMIC-dependent exons by other RBPs in fly brains; data are from (*40*). In red/blue, number of exons regulated in the same/opposite direction between each RBP and the eMIC domain at two ΔPSI levels. (**B**) Effect of the double knockout of *elav* and *fne* on exon inclusion (PSI) genome-wide in the first-instar larval (L1) CNS. Data are from (*41*). Exons are grouped on the basis of their response to eMIC insufficiency (see Materials and Methods). PSI frequency distributions of eMIC-dependent exons in control and mutant CNS are depicted in green. (**C**) Overlap between eMIC-dependent exons, neural-enriched exons, and Elav/Fne up-regulated exons. The stage of the samples used for the definition of each exon group is indicated. (**D**) Sashimi plot of RNA-seq data from L1 CNS upon KD of *elav* and *fne*. Pink arrows indicate putative binding motifs of Elav. Data are from (*41*). (**E** and **F**) RT-PCR assays of *Srrm234* terminal exons from wild-type (wt) and *elav*-hypomorphic (*elav*^edr^) larval eye imaginal discs (J) and from SL2 cells upon overexpression of Elav (K). Blue triangles mark primer positions. The main splicing products corresponding to annotated isoforms from the *Srrm234* gene (A, C/F, and G) are labeled. Nonlabeled bands correspond to intermediate splicing products or unspecific amplification that do not differ substantially between samples. (**G**) Summary of the main regulatory interactions identified involving the eMIC splicing program in *Drosophila*.

RBPs often cross-regulate each other and co-regulate the splicing of individual alternative exons (*34*, *38*, *39*). Hence, we searched for trans-acting factors whose regulatory programs may overlap with eMIC-dependent splicing by analyzing a recent dataset on RBP KDs on fly brains (Fig. 7A and fig. S8D) (*40*). Among all factors, the RBP with the largest overlap with eMIC targets was *pasilla* (ps), the mammalian *Nova1/2* ortholog (Fig. 7A). This effect is likely due to a direct role in co-regulating eMIC-dependent exons and not indirectly through regulation of eMIC domain expression since neither *ps* nor any other RBP KD from this dataset altered the AS at the 3′ end of *Srrm234* (fig. S8E).

In contrast, analysis of a recent dataset from first-instar larval (L1) CNS where two members of the ELAV family were knocked out (*41*) gave very different results. *Embryonic lethal abnormal vision* (Elav) is an RBP widely used as a neuronal marker that, among other functions, can regulate neuronal AS and APA, and have redundant roles with its paralog *found in neurons* (Fne) (*41*–*43*). The majority of eMIC-dependent exons were completely skipped in L1 CNS upon double depletion of *elav* and *fne* (Fig. 7B), and the splicing programs of Elav/Fne and the eMIC domain widely overlapped (*P* = 8.4 × 10^−142^ hypergeometric test; Fig. 7C). In this case, the major source of the overlap seemed to be the regulation of *Srrm234* last exon selection by the overlapping function of Elav and Fne (Fig. 7, D to F, and fig. S8, F to H), although a direct effect on some eMIC targets cannot be ruled out. Besides, we identified several putative binding sites for Elav (UUUNUUU motifs) in the 3′ end of *Srrm234* (Fig. 7D). Consistently, these two proteins promoted not only the skipping of the proximal poison exon at the 3′ end of *Srrm234* (Fig. 7D) but also the selection of the distal poly-A site (fig. S8F). Through RT-PCR assays of the 3′ end of *Srrm234* transcripts in *elav*-hypomorph (*elav*^edr^) (*44*) eye imaginal discs, we found that *elav* insufficiency prevented the skipping of the poison exon also at this stage and tissue, leading to higher isoform G expression and the complete absence of eMIC expression (Fig. 7E). Moreover, heterologous expression in SL2 cells showed that Elav alone is sufficient to promote eMIC expression (Fig. 7F and fig. S8G), and iCLIP (cross-linking and immunoprecipitation) data from fly heads from a recent study (*42*) suggest direct binding of Elav to this region of the *Srrm234* pre-mRNA (fig. S8H).

In summary, we found that the eMIC splicing program is recruited to neural tissues by the Elav/Fne-mediated regulation of *Srrm234* 3′ end processing and that, within this tissue, it modestly overlaps with splicing networks regulated by other RBPs (Fig. 7G). We identified several RBPs and transcriptional regulators with alternative isoforms misregulated upon eMIC insufficiency (Fig. 7G and table S3), placing the eMIC splicing program within a dense network of neural gene expression regulation, similar to previous results from mammalian model systems (*45*).

### The *Drosophila* eMIC splicing program shapes the repertoire of neuronal ion channels

**Fig. 8.**
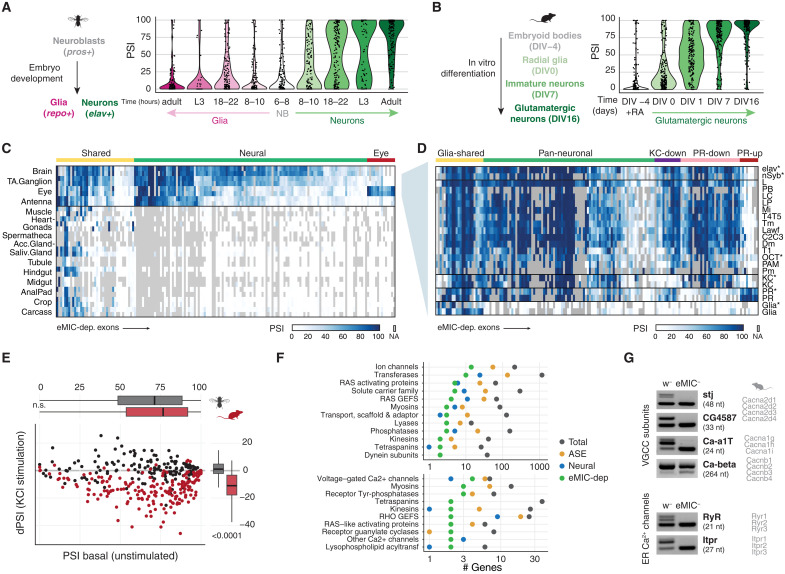
Landscape of the eMIC splicing program across tissues and neural cell types. (**A**) Inclusion levels (PSI) of eMIC targets in neuronal and glial cell populations at different times of embryo development and larval and adult samples. Data sources in table S1. NB, neuroblasts; L3, third-instar larvae. (**B**) Inclusion of mouse eMIC targets along an in vitro differentiation time course from embryoid bodies to glutamatergic neurons. Data are from (*76*). DIV, days from the onset of differentiation; EB, embryoid bodies; NPC, neural precursor cells. (**C**) Heatmap of the inclusion levels of eMIC-dependent exons across adult tissues. TA.Ganglion, thoracicoabdominal ganglion; Acc.Gland, male accessory glands; Saliv.Gland, salivary glands. Data from the FlyAtlas 2 and others (table S1). (**D**) eMIC exon inclusion levels in different neuronal types. Data are from (*48*), unless marked with an asterisk (table S1). KC, Kenyon cell; PR, photoreceptor. Groups are based on the inclusion profile across neural cell types (see Materials and Methods for definitions). (**E**) Effect of KCl-induced neuronal depolarization on the inclusion of eMIC-dependent exons. *Drosophila* data are from (*52*), and mouse data are from (*13*). *P* values are from Mann-Whitney *U* tests. (**F**) Gene groups with more than one member bearing an eMIC-dependent exon. Top: “Top-level” gene categories. Bottom: Specific gene subgroups. ASE, other alternatively spliced exons. (**G**) RT-PCR validations of eMIC-dependent exons in calcium channels from w^−^ and eMIC^−^ heads. On the right, mouse orthologous genes. VGCC, voltage-gated calcium channel; ER, endoplasmic reticulum.

In line with the Elav-driven expression of the eMIC domain, inclusion of eMIC exon targets increased progressively during embryonic development in neurons but not in glial cells, similar to the pattern observed for *Srrm3/4* targets in mouse (Fig. 8, A and B) (*17*). Consistent with this observation and with the large overlap with the neural-specific AS program (Fig. 6F), the vast majority of eMIC-dependent exons were enriched in all neural tissues: brain, eye, antenna, and thoracicoabdominal ganglion (Fig. 8C). Still, 15 eMIC-dependent exons were specifically enriched in the eye, including exons in the ion channels *Otopetrin-like a* (*OtopLa*) and *Chloride channel a* (*ClC-a*), the kinase *retinal degeneration A* (*rdgA*), *crumbs* (*crb*), a key regulator of Notch activity via the Hippo pathway, and its interacting partner *karst* (*kst*). We found that most eMIC targets were expressed at higher levels in the adult and L1 larval CNS when compared to L3 larval CNS (fig. S9, A to C), likely reflecting the higher proportion of immature neurons in L3 CNS when compared to L1 CNS (*46*, *47*).

Next, we profiled eMIC-dependent exon expression across cell types in neural tissues using a recent RNA-seq dataset of cell types in the fly optic lobe (*48*) as well as other datasets of sorted neurons (Fig. 8D; fig. S9, D to H; and table S1) (*49*–*51*). Given that the sequencing depth of some of these samples was too low to robustly quantify exon inclusion and that eMIC exon inclusion profiles were very similar among closely related cell types (fig. S9D), we merged related samples to obtain better estimates of exon inclusion (Fig. 8D and fig. S9E). These data revealed several clear patterns. First, we confirmed the neuronal specificity of the splicing program regulated by the eMIC domain, with very few eMIC exons being expressed also in glial cells (Fig. 8D and fig. S9F, “glia-shared” exons). Second, eMIC exons were broadly included across neuronal types but with some degree of variability, with photoreceptors showing the most divergent eMIC exon profile (Fig. 8D). As expected, the most photoreceptor-enriched eMIC targets largely overlapped with the set of eye-enriched exons (fig. S9F), indicating that the eye signature mainly stems from the photoreceptor population. In addition, a fraction of eMIC exons was specifically depleted in photoreceptors and a smaller set was depleted only in Kenyon cells (Fig. 8D). These patterns were mirrored by the pan-neuronal expression of the eMIC domain across cell types (fig. S9H) and by the photoreceptor-specific expression of the newly identified eye-specific *Srrm234* 3′ end isoform (Fig. 1C and fig. S9H). This variability of the eMIC splicing program across neuronal types followed the same trend as that of all alternatively spliced exons genome-wide (fig. S9, E and G).

We then looked at the interplay between the eMIC splicing program and transcriptomic signatures of sustained neuronal activity. Mouse Srrm4-regulated exons were shown to decrease inclusion upon sustained KCl-induced neuronal depolarization (*13*). To study whether this connection is also present in *Drosophila*, we quantified eMIC exon inclusion using a published dataset of fly brains treated with KCl or activated through optogenetic stimulation (*52*). Unlike the observed effect in mouse, *Drosophila* eMIC exons did not change their inclusion levels upon KCl treatment or optogenetic stimulation (Fig. 8E and fig. S10A). Nonetheless, the behavioral and physiological phenotypes associated with eMIC insufficiency in flies suggested brain-wide alterations in neuronal activity (Fig. 5). Thus, we used these RNA-seq datasets to derive a list of genes up-regulated upon KCl or optogenetic stimulation taking into account both end and intermediate time points (activity-regulated genes; fig. S10B) (*52*). We found that this set of genes is overrepresented among the differentially expressed genes in eMIC^−^ brains (7 of 211, *P* = 2.4 × 10^−4^, Fisher’s exact test; fig. S10C and table S4), further supporting the dysregulation of neuronal activity in these mutants.

To dig into the molecular functions of genes containing eMIC-dependent exons, we next used the gene group classification from FlyBase (Fig. 8F). Gene group classification follows a hierarchical organization, and thus, we focused on both the top and bottom levels, i.e., broad and specific groups, respectively. At the top level, the most numerous category corresponds to ion channels, with 14 genes hosting eMIC exons annotated in this group (Fig. 8F, top). Looking at the most specific gene families and complexes, two groups of calcium channels were overrepresented. First, four of seven subunits forming *Drosophila* voltage-gated calcium channels have eMIC-dependent exons: *stj*, *CG4587*, *Ca-*α*1T*, and *Ca-*β (Fig. 8, F and G). Second, two of the three main intracellular calcium channels are alternatively spliced in an eMIC-dependent manner: the ryanodine and inositol-3-phosphate receptors, *RyR* and *Itpr* (Fig. 8, F and G). These splicing alterations on ion channels, in general, and calcium channels, in particular, may underlie the altered neuronal activity in eMIC^−^ larvae unveiled by our brain imaging experiments.

### Parallel evolution of the fly and mammalian eMIC splicing programs controlling neuronal physiology

**Fig. 9.**
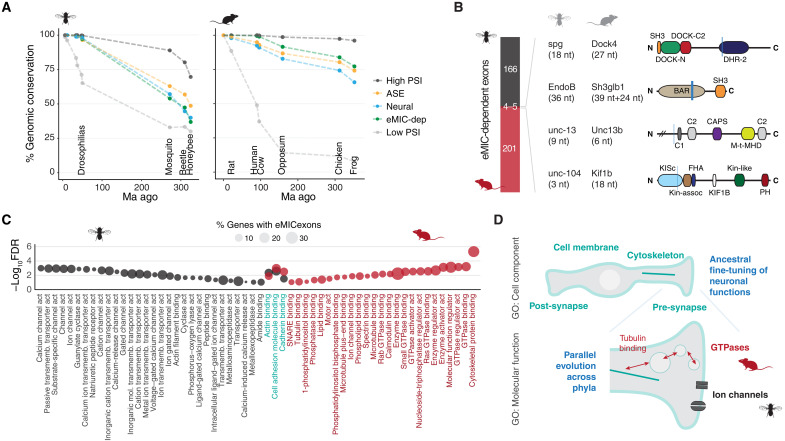
Evolution of the eMIC splicing program in flies and mammals. (**A**) Conservation of eMIC-dependent exons at the genome level based on liftOver. (**B**) Overlap between the mouse and fly eMIC splicing programs. For the four shared exons, gene name and exon length are indicated in each species. On the right, exon positions are marked within the protein domain scheme with a vertical blue line. (**C**) Gene ontology (GO) terms in the “Function” category that are enriched in *Drosophila* and mouse eMIC targets (black and red, respectively). Terms enriched in both species are in blue. Circle size represents the percentage of eMIC exon-bearing genes in each GO category. (**D**) Summary schematic representation of GO terms enriched in both *Drosophila* and mouse eMIC targets in the “cellular component” category (top) and of species-specific terms in the “molecular function” category (bottom). GTPases, guanosine triphosphatases.

The old ancestry of the neural microexon program and the availability of perturbation data from distantly related species make it an appealing case study for the evolution of splicing networks. Hence, we investigated the extent of conservation between the fly and mammalian eMIC-dependent splicing programs. In line with previous studies (*11*, *15*), ~75% of mouse *Srrm3/4* targets were conserved across tetrapods at the genomic level (*53*) (Fig. 9A and fig. S11, A to C). On the other hand, *D. melanogaster* eMIC exons were highly conserved within the *Drosophila* genus but showed little conservation with other holometabolous insects, such as *Anopheles gambiae* (mosquito), *Tribolium castaneum* (the red flour beetle), and *Apis mellifera* (honey bee) (Fig. 9A and fig. S11, D to F). Moreover, fly eMIC exons shared with other holometabolous insects were not always present in closer related species (fig. S11F), highlighting a higher evolutionary turnover of the eMIC splicing program within this clade compared to vertebrates. Nevertheless, we identified high conservation in the flanking intronic sequences of *Drosophila* eMIC targets similar to those of other alternatively spliced exons in this species (fig. S11G), in line with the proposed regulatory role of these genomic regions. This high conservation was also described for the mammalian eMIC exon program, which showed a particularly high conservation in this region, even when compared to other alternatively spliced exons (fig. S11G) (*11*).

Of the 157 genes with eMIC exons in *D. melanogaster*, only 19 of them had a mouse ortholog bearing an Srrm3/4-regulated exon, and, of these, only four exons were in the same position as in the fly orthologous gene: *sponge* (*spg*), *Endophilin B* (*EndoB*), *unc-13*, and *uncoordinated-104* (*unc-104*) (Fig. 9B and fig. S11H). Moreover, only the exon in *EndoB* could be identified in the genomes of all other studied insect species, favoring a scenario of convergent evolution rather than of common ancestry for the remaining shared eMIC exons. Notably, these results thus indicate that eMIC splicing programs have been nearly completely rewired since their common origin in bilaterian ancestors.

Given this low level of conservation between phyla, we then wondered whether the eMIC domain affects similar or divergent biological processes in flies and mammals. To avoid biases introduced by gene ontology (GO) annotations in different species, we based our analysis on the more comprehensive human annotation and performed enrichment analyses of the mouse and fly eMIC targets using GO categories transferred from the human orthologs (see Materials and Methods for details). Some GO terms were enriched similarly in mouse and *Drosophila* eMIC targets, indicating a shared bias for genes present in the plasma membrane, cell projections, and the synapse, as well as for cytoskeletal proteins (Fig. 9C and fig. S12A). However, most enriched categories were only observed for targets of a single species. The most notable case was the contrasting enrichment for ion channels in the *Drosophila* program and for guanosine triphosphatases (GTPases) in mouse (Fig. 9C). Similar results were also obtained using fly GO annotations as reference (fig. S12, B and C) or swapping background gene lists. These results thus suggest that, since the eMIC domain originated in their last common ancestor, each phylum has independently assembled splicing programs that control distinct molecular modules within neurons, which nonetheless ultimately modulate neuronal excitability (Fig. 9D).

## DISCUSSION

### Control of neuronal physiology by the eMIC splicing program in *Drosophila*

The availability of RNA-seq datasets across fly tissues and developmental stages has uncovered hundreds of splicing decisions that shape their transcriptome and proteome (*5*, *54*). However, contrary to vertebrate model organisms, most mechanistic studies have focused on the characterization of a handful of individual splice isoforms, lacking a portrait of how the controlled perturbation of broader splicing programs affect physiology. Here, we generated transgenic lines for *Srrm234* isoforms and a loss-of-function mutant for the eMIC domain, responsible for the regulation of neuronal microexon programs across Bilateria (*15*). We showed that this domain is encoded as an alternative isoform of the pan-eukaryotic *Srrm234* locus that is expressed in neurons due to regulated 3′ end processing by Fne and Elav. These two RBPs have prominent roles on RNA metabolism in neurons and are widely conserved across metazoans (*55*, *56*), making it good candidates for restricting eMIC expression to neurons also in bilaterian ancestors. In *Drosophila*, we further show that ~28% of Elav/Fne positively regulated exons and one-third of all neural-enriched exons depend on the eMIC domain for their inclusion, acting as a master regulator of neuron-specific AS.

Our genome-wide analysis across neural cell types has highlighted a shared pan-neuronal AS program, with the notable exception of photoreceptors. On a finer level, Kenyon cells in the mushroom body also have a unique splicing signature that is uniform across different studies (*48*, *50*). Cell type–specific characterization of the eMIC-regulated splicing program alone mirrored these general AS patterns, placing it as a marker of pan-neuronal identity that nevertheless overlaps with other programs controlling neuron type–specific AS. A recent study has showed that *Srrm234* (CG7971) expression in mushroom body neurons is required for ethanol-cue–induced memory (*57*). Besides, a different study has identified cycling behavior of *Srrm234* transcripts in dorsal lateral neurons, potentially connecting this program with the circadian clock (*58*). Here, we show that the splicing alterations in the *Srrm234* eMIC-specific mutant result in an array of neurological-associated phenotypes, most evident of which are locomotion alterations. These, together with the above studies and our brain imaging, sleep, and bang sensitivity results, suggest widely pleiotropic effects for this splicing program across the fly nervous system, which could be at least partly explained by the enrichment of eMIC-dependent exons in ion channels and their role in neuronal excitability and function across neuronal populations.

Mis-splicing of ion channels could also underlie the deleterious effect of ectopically expressing the eMIC domain outside the nervous system (*59*), as we describe here for the wing. In addition, other genes with prominent roles in neuronal function and development are alternatively spliced in eMIC^−^ brains, including genes in key signaling pathways such as Notch (*sno*, *scrib*, *shrb*, and *Tsp26A*), BMP (*sax*), Wnt (*spen*), NK-kB (*LRR*), EGFR (*Ptp10D*, *Ptp4E*, *RasGAP1*, *spen*, and *Src42A*), and Hippo (*jub* and *crb*) (table S3). The transgenic constructs generated for *Srrm234* will thus be valuable to dissect the function of this splicing program and the relevance of spatially restricting its expression. Note, however, that our rescue experiments within neurons exposed that quantitative and spatial regulation of eMIC expression is particularly sensitive. Hence, more elaborated genetic perturbations might be necessary to fully recover the complex regulation of the *Drosophila Srrm234* locus (fig. S1) and its function on splicing regulation beyond the eMIC domain, as in the case of the Cwf21 domain in *Srrm234* and the regulation of *Dscam* exon 9 cluster (*60*).

### Evolution of neuronal eMIC-regulated programs in flies and mammals

Comparative transcriptomics across metazoans showed that neuronal microexons originated in bilaterian ancestors driven by the appearance of the eMIC domain and that neuronal microexons are largely shared within vertebrates (*11*, *15*). However, conservation rapidly declines outside this group, even in the cephalochordate amphioxus, similar to previous reports on Nova-regulated exons (*61*). Here, by characterizing the full AS landscape regulated by the eMIC domain in *D. melanogaster*, we have confirmed the small overlap between the fly and mammalian programs, with only four exons in equivalent positions within the orthologous genes. Moreover, despite the high conservation within the *Drosophila* genus, eMIC-dependent exons in holometabolous insects show a fast rate of evolution compared to that in vertebrates, with only nine *Drosophila* exons present in all three non-drosophilid insects studied (*A. gambiae*, *T. castaneum*, and *A. mellifera*). Notwithstanding, 18 additional eMIC-dependent exons in *Drosophila* and 23 in mouse are present within orthologous genes but at different positions. This recurrence of alternative exons within orthologous genes regulated by the same splicing factor has been previously seen for the *epithelial splicing regulatory protein* (Esrp)–regulated splicing programs across deuterostomes (*62*) and, more recently, for *Nova*-regulated exons between *Drosophila* and mouse (*63*), suggesting the presence of hotspots for the evolution of new exons as a common feature in the evolution of splicing programs.

Despite the extensive rewiring of its target exons, the eMIC domain has been associated with neuronal fate expression since its origin in bilaterian ancestors as an alternative isoform of *Srrm234* (*15*). This AS event brought new regulatory capacity to an ancestral regulator similarly to other described cases for transcriptional and splicing regulators [e.g., (*64*, *65*)], ending up in the parallel reassembly of AS programs controlling neuronal function that deploy different modules of the neuronal toolkit in different clades.

## MATERIALS AND METHODS

### Generation of *Srrm234*^eMIC−^ mutant line

The eMIC-null allele for *Srrm234* (*CG7971*) was generated by GenetiVision CRISPR gene targeting services. The 650–base pair (bp) deletion at the 3′ end of the gene was generated using gRNAs *aggtcaaccaaggcggggc* and *gactccggctgttgcgcag* together with donor template harboring two homology arms flanking a *loxP 3xP3-GFP loxP* cassette (fig. S2D). Left and right homology arms of the donor were amplified using primers CG7971-LAF3 and CG7971-LAR3 and CG7971-RAF4 and CG7971-RAR4, respectively. Successful deletion and integration of the cassette was validated by PCR and Sanger sequencing using primers CG7971-outF3 and CG7971-outR4 and LA-cassette-R and Cassette-RA-F, respectively. All primer sequences are included in table S5. For microscopy experiments, the *3xP3-GFP loxP* cassette was excised by crossing our mutant line with a line expressing Cre-recombinase under a heat-shock inducible promoter.

The deletion at the 3′ end of *Srrm234* removes the two possible terminal exons of the gene and adds an additional splicing acceptor and poly-A signal to ensure proper transcriptional termination (Fig. 2A and fig. S2D). This mutation thus generates a C-terminal truncation after the second-to-last exon, mainly affecting the coding region of eMIC-containing isoforms but also removing the last four amino acids of isoforms A/G. This mutant line has a milder phenotype compared to a full gene knockout generated by CRISPR-deleting the 5′ end of the gene (encompassing all annotated promoters), which is embryonic lethal (*60*).

### Generation of transgenic *Drosophila* lines

Transgenic lines expressing Srrm genes under a 5xUAS promoter were generated at the Francis Crick Institute fly facility. Drosophila Srrm234-A and Srrm234-C isoforms and human SRRM4 open reading frames were subcloned from available vectors (*15*). With the aim of expressing the eMIC domain in the most physiological way possible, we also cloned an additional isoform of Srrm234 that includes a protein-coding intron retention event that is almost 100% retained in neural tissues (fig. S1), which we termed isoform “I.” This intron retention event is present in the annotated isoform F, but the full-length isoform that we cloned from brain cDNA lacked the additional exon at the 5′ end of isoform F; thus, a new name was given to avoid conflicts with the current gene annotation. All constructs bear an N-terminal 3xFlag tag, were verified by Sanger sequencing, cloned into vector pUASTattB, and integrated at sites attP40 in Chr2 or attP2 in Chr3, using a line expressing Phi31 integrase from ChrX. Positive integrants were balanced accordingly to maintain the stock.

### *Drosophila* strains and culture

Flies were maintained at 25°C in 12-hour light/12-hour dark conditions. A list of the published and unpublished stocks used can be found in table S5.

### Cloning of *Srrm234* 3′ end minigene and Srrm234 protein isoforms

To generate the *Srrm234* 3′ end minigene, the genomic region encompassing the last three exons and corresponding two introns (chromosomic region chr3L:1,654,247-1,655,460) was amplified from *D. melanogaster* genomic DNA and cloned in pAc vector. PT1 and PT2 sequences were added upstream to the first exon and downstream to the last exon, respectively (*66*). In addition, a mutation was performed in the last exon (from nucleotide 148 to nucleotide 164) to introduce an Sp6 motif for detection of the pattern of AS independently of the endogenous *Srrm234* transcripts. In the case of the tested Srrm proteins, they were cloned in pAc vector with N-terminal epitopes. pAc vector was a gift from F. Gebauer’s laboratory. Srrm234 protein isoforms (A, C, I, and E) bear a T7-3xFlag tag, Elav, a 3xFlag tag, and human SRRM4, a T7 epitope. All primer sequences used for cloning are listed in table S5.

### Transfection in Schneider S2 cells

A total of 400,000 Schneider SL2 cells were transfected with 50 ng of plasmid bearing the Srrm234 minigene (when it applies) and 3 μg of Srrm protein expression plasmid (or control) using Lipofectamine 2000 (Invitrogen, following the manufacturer’s instructions) and plated in six-well plates. Cells were collected 48 to 72 hours after transfection. RNA was extracted using RNAspin Mini Kit (GE Healthcare).

### Quantification of gene expression and exon inclusion

For head extractions, flies were snap-frozen in liquid nitrogen and mechanically dissociated by brief vortexing. Heads were separated from the rest of body parts using a mini-sieve system precooled in liquid nitrogen, and stored at −80°C. Larval wing discs were dissected in cold phosphate-buffered saline (PBS) and stored on RNAlater. RNA was extracted using NZY Total RNA Isolation kit (MB13402), and cDNA was generated from 250 ng of RNA per sample using SuperScript III (Thermo Fisher Scientific) and anchored oligo-dT primers, following the manufacturer’s instructions. RT-PCR assays of alternatively spliced exons were done using primers annealing to the flanking upstream and downstream constitutive exons. Expression levels of *Srrm* constructs were assessed by quantitative PCR using NZYSpeedy One-step RT-qPCR system and detected with a Roche LightCycler 480 instrument. All primer sequences are provided in table S5.

### Western blot analysis

Proteins were extracted from SL2 cells pellets using radioimmunoprecipitation assay buffer [20 mM tris-HCl (pH 7.5), 150 mM NaCl, 1 mM EDTA, 1% Triton X-100, 0.1% SDS, 1 mM dithiothreitol, and proteinase inhibitors (Roche)]. After quantification using BCA (Thermo Scientific), proteins were separated on a 12% SDS-polyacrylamide gel and transferred to a nitrocellulose membrane (GE Healthcare). Tris-buffered saline and Tween 20 (TBST) supplemented with 5% milk was used for blocking and antibody incubation. The following antibodies were used: Elav (Developmental Studies Hybridoma Bank) and horseradish peroxidase (HRP) anti-rat (Dianova). Membranes were incubated in Western blot highlighting Plus ECL solution (PerkinElmer Inc.) before image acquisition using the iBright system. Membranes were stained using Ponceau (Sigma-Aldrich) as a loading control.

### Weight quantification and longevity assay

Flies younger than 1 day were snap-frozen in liquid nitrogen and weighted in groups of five using a micro-balance with a detection limit of 0.1 mg. Results were provided as average weight by dividing each measurement by 5; a total of 50 flies per genotype were measured. *P* values for the difference between genotypes were calculated using Student’s *t* tests.

The life span at 25°C of 50 to 100 flies for each sex and genotype was monitored every 2 days, when flies transferred to a new food vial. Flies that escaped during the transfer were right-censored. *P* values for the difference in survival were calculated using the log-rank test.

### Negative geotaxis assay

We separated 3-day-old males and females in groups of 10 flies 24 hours before the experiment. We transferred the flies to dry 50-ml serological pipettes and let them habituate for 2 to 3 min. We video-recorded for 1.5 min after tapping the cylinders. We manually counted the number of flies crossing each 5-ml mark (corresponding to 2.5-cm intervals) for each second during 30 s after tapping. We repeated the experiments at least three times (30 flies) and calculated mean height positions at each second and nonparametric 95% confidence intervals with 10,000 bootstrap replicates.

### Bang sensitivity

Three-day-old male and female flies were separated in groups of 10 individuals 24 hours before the experiment. On the day of the experiment, flies were transferred to clean and dry vials, which were vortexed at a maximum speed for 10 s. Each vial was recorded for 2 to 5 min. Recorded videos were manually inspected, the number of paralyzing flies was quantified, and differences between genotypes were assessed with Fisher’s exact tests. The time until recovery (i.e., when flies were to upright position) was also documented. Flies that did not recover during the duration of the recorded video were right-censored. Statistical differences in recovery time were calculated using Mann-Whitney *U* tests.

### Sleep and daily activity patterns

Male and female flies were monitored using TriKinetics *Drosophila* Activity Monitors, which measure the number of times each individual fly crosses a laser beam (activity counts), using 1-min bins. Each fly was placed into a glass tube containing 2% agarose and 5% sucrose food. Flies were entrained for 4 days in 12-hour light/12-hour dark cycles. All the experiments were performed at 25°C. The sleep and activity parameters were analyzed using the MATLAB script SCAMP (https://academics.skidmore.edu/blogs/cvecsey/files/2019/03/Vecsey-Sleep-and-Circadian-Analysis-MATLAB-Program-SCAMP-2019_v2.zip). Activity counts were averaged across 3 days after entrainment for each individual fly. We assayed 16 to 32 flies per sex and genotype at two ages: recently hatched (3 days old) and middle age (21 days old). Statistical differences in maximum sleep duration and total number of activity counts between genotypes were assessed using Mann-Whitney *U* tests.

### Larval locomotion analysis

For each experiment, five third-instar larvae were placed at the center of a 10-cm petri dish with 1% agarose and allowed to crawl freely. Dishes were placed on an artist light panel to provide enhanced illumination and recorded from the top at 30 frames per second. Larvae were tracked using BIO (https://joostdefolter.info/bio-research), and output data of larval contours and centers of mass were used for subsequent analysis. Larvae were tracked within a restricted field of view. If a larval contour touched its perimeter (i.e., the larva was exiting this field of view), the larva was not tracked from this time point onward. Where larvae collided, a reliable contour and center of mass could not be computed; therefore, data during collision were excluded. Larval speed was calculated as the Euclidean distance between the centers of mass of consecutive frames. Mean speed (in pixels per frame) were converted to millimeters per second by calibrating pixel values to real world values based on the diameter of the behavioral area.

Larval trajectory straightness was determined using the R package “trajr” (*67*). Centers of mass and time values for each larva were used to create a trajr trajectory object. Following this, path straightness was determined using the TrajStraightness function, which approximates the efficiency of a directed walk. Straightness indices range between 0 (infinitely tortuous) and 1 (a perfectly straight line). Larval curved body axis was computed using the larval contour output from BIO. As a larva curls into a tighter ball, its contour approximates a circle, which can lead to complications with precise head-tail assignment. Total contour length was calculated as the total Euclidean distances between all contour points. Primary axis circumference was calculated by multiplying the larval primary axis length (defined as being the largest Euclidean distance between pairwise contour points) by pi. A “ratio of curvature” was computed per frame as being the total contour length/primary axis circumference. A ratio of 0.9 and above was taken to indicate larval curling and was used as a threshold to determine the time that each larva spent curled.

### Fitness test

We set up F0 crosses using always females of the same genotype and crossing them to males of different genotypes. We quantified the number of flies in the F1 generation on the basis of their sex and the presence of the Sb^−^ marker from the TM3 balancer chromosome. For each genotype and sex, we calculated their relative fitness values as the ratio between their observed allele frequencies and the expected frequencies derived from the Mendelian ratios coming from the mating schemes (0.25:0.25:0.25:0.25). For each cross, we quantified a total number of F1 flies of at least 2500. Statistical differences in the allele frequencies for each cross were assessed using chi-square tests for the average number of flies per replicate.

### Immunostaining

Immunostaining with mouse anti-Fas2 1D4 and anti-brp (nc82) antibodies (Developmental Studies Hybridoma Bank) was performed at 1:50 and 1:20 dilutions (respectively) using standard protocols, followed by anti-mouse-Alexa Fluor 647 (Thermo Fisher Scientific) or anti-mouse-Cy5 (Jackson ImmunoResearch) secondary AB staining, at 1:200 and 1:100, respectively. Nuclei were stained with 4′,6-diamidino-2-phenylindole (DAPI), captured with a Leica SP5 confocal microscope, and processed with Fiji (*68*).

For NMJ staining, third-instar larvae were dissected in cold PBS and fixed with 4% paraformaldehyde in PBS for 45 min. Larvae were then washed in PBS-T (PBS + 0.5% Triton X-100) six times for 30 min and incubated overnight at 4°C with mouse anti-synaptotagmin, 1:200 (3H2 2D7, Developmental Studies Hybridoma Bank). After six 30-min washes with PBS-T, secondary antibody anti-mouse conjugated to Alexa Fluor 488 and tetramethyl rhodamine isothiocyanate–conjugated anti-HRP (Jackson ImmunoResearch) were used at a concentration of 1:1000 and incubated at room temperature for 2 hours. Anti-HRP is used as a marker to stain neuronal membranes in insects (*69*). Larvae were washed again six times with PBS-T and lastly mounted in VECTASHIELD. Images from muscles 6 to 7 (segment A2-A3) were acquired with a Leica Confocal Microscope SP5. Serial optical sections at 1024 × 1024 pixels with 0.4-μm thickness were obtained with the ×40 objective. Bouton number was quantified manually using Imaris 9 software.

### Calcium imaging experiments

Feeding third-instar larvae were dissected in physiological saline composed of 135 mM NaCl, 5 mM KCl, 5 mM CaCl_2_-2H_2_O, 4 mM MgCl_2_-6H_2_O, 5 mM 2-[[1,3-dihydroxy-2-(hydroxymethyl)-propan-2-yl]amino]ethanesulfonic acid, and 36 mM sucrose, adjusted to pH 7.15 with NaOH. To prepare the sample for light sheet microscopy, the isolated CNS was embedded in 1% UltraPure Low Melting Point Agarose (Thermo Fisher Scientific) in physiological saline at 36°C and mounted in a glass capillary (inner diameter, 1.4 mm; outer diameter, 2 mm) as previously described (*26*). Once cooled, the agarose was pushed out to expose the embedded CNS outside of the glass capillary. The sample was placed in the imaging chamber filled with physiological saline, and spontaneous changes in GCaMP7b intensity were recorded as a proxy for neuronal activity in vGlut-GAL4–expressing neurons in the VNC. Using a Luxendo-Bruker MuVi-SPIM, volumes of either 11 or 22 slices taken every 5 to 7 μm were imaged with a 4.1-μm light sheet at 0.47 Hz, with an exposure of 20 or 2 ms per frame, respectively, and a delay time of 11 ms. Images of 2048 × 2048 pixels were taken using a ×16 objective.

Only dorsal views were saved and processed manually in Fiji (https://imagej.net/Fiji): Images were binned 3 × 3 in *XY* using BigDataProcessor (*70*), and maximum intensity projections were motion-corrected using StackReg (*71*). Following background subtraction, mean intensities for each half-segment, from the first thoracic to the eighth or ninth abdominal segment, were obtained using the Multi Measure tool in ROI Manager. Values were imported into MATLAB (R2019b, MathWorks) and Δ*F*/*F* = (*F*(*t*) − *F*_0_)/*F*_0_ traces were calculated, where *F*(*t*) is the fluorescence intensity at a given time point and *F*_0_ is the mean fluorescence intensity in a manually defined window lacking any spontaneous activity, spanning 10 frames (~21.3 s).

To identify activity bouts, Δ*F*/*F* data were averaged across segments and smoothened using a low-pass filter, and local maxima were identified using the “findpeaks” function in MATLAB. Smoothened traces were obtained using a short-time Fourier transform (FT), followed by an inverse FT with a low-pass filter. Briefly, data were split into fragments of 100 frames (~3.5 min) overlapping by ½, each fragment was windowed using a Hamming function, its discrete FT was computed, and the resulting magnitudes and phases were used to reconstitute the data from the five lowest frequency components.

To identify the number of waves and their mean amplitudes, Δ*F*/*F* data were averaged across segments and findpeaks was used to extract the locations and amplitudes of local maxima in each sample. Peak amplitudes were measured from baseline. Next, Δ*F*/*F* data from three frames flanking each peak location (resulting in seven frames = 14.9 s) were extracted from each segment, and the location of the maximum peak was identified, together with its width at half-height. For each wave, the slope of a linear fit for peak locations as a function of VNC segment was used to determine its type, with a negative slope identifying a forward wave and a positive slope identifying a backward wave. Peak widths were used to determine the starting and ending points of a wave.

One-sided activity was assessed by measuring absolute differences in intensity between each left-right segment pair. Values represent the percentage of recording time in which the absolute difference is larger than a predefined threshold of 0.7 Δ*F*/*F* on average per sample. The threshold was established by comparing visually observed one-sided activity to noise in two example samples. MATLAB scripts are available at https://github.com/PrietoGodinoLab/eMIC_GCaMP.

### Single-cell RNA-seq

Fifty whole CNSs (brain lobes and VNC) were dissected from L1 larvae 1 to 2 hours after hatching for control w^1118^ and eMIC^−^ larvae in ice-cold Schneider’s medium and transferred into an embryo dish with 1× DPBS on ice. Total dissection time did not exceed 2 hours. After dissection, samples were transferred into 0.5-ml vials containing dissociation solution [DPBS with papain (0.2 U/ml), collagenase (0.1 mg/ml), and 0.04% bovine serum albumin (BSA)]. Brains were briefly centrifuged at 4°C, resuspended in 80 μl of fresh dissociation solution, and left dissociating for 30 to 40 min at 25°C shaking at 1000 rpm. Every 5 min, tissue samples were triturated using a 200-μl pipette tip (50 to 80 times). After dissociation, cells were passed through a 20-μm Flowmi cell strainer and centrifuged at 2000 rpm for 5 min at 4°C. Dissociation solution was removed and substituted by DPBS + 0.04% BSA. A total of 10 μl of dissociated cells was used to determine cell yield by using Hoechst staining under a fluorescent stereomicroscope and a C-Chip Neubauer. Samples were adjusted at a concentration of 1200 cells/μl for loading into Chromium single-cell chip. Single-cell RNA-seq libraries were prepared using the Chromium Single Cell 3′ Library and Gel Bead Kit v3 according to the manufacturer’s instructions. Sequenced libraries were processed using Cell Ranger v2.0.0 provided by 10x Genomics, resulting in 29,554 cells with 1232 unique molecular identifiers (UMIs) and a median of 693 genes per cell for control sample and 29,942 cells with 1205 UMIs and a median of 679 genes per cell for the eMIC^−^ mutant sample.

### Single-cell RNA-seq analysis

Sequencing data were analyzed using Seurat 4.0.0 R package. Cells with less than 5% mitochondrial genes and a unique feature count between 50 and 2500 were selected for downstream analysis, leaving 19,400 cells in the control and 18,268 cells in the mutant sample. Data were normalized with a log scale factor of 10,000, and 2000 highly variable features were selected according to the R package’s developer recommendation. After inspection of Elbow plots, Jackstraw plots, and principal component heatmaps, 20 principal components were selected to explain the variability of the data. To subdivide the datasets into clusters, the Seurat command FindNeighbors with an HD.Dim of 20 and FindClusters with a resolution of 0.5 were used. Last, the dataset with reduced dimensionality was visualized using a UMAP (uniform manifold approximation and projection) plot, which led to the generation of 16 clusters in both the control and the mutant datasets. Data integration for control and mutant samples was performed by identifying the variable features present in both and selecting them for integration. Within the integrated data, common cell types were identified on the basis of known marker genes from visualizations using the FeaturePlots Seurat.

### Bulk RNA-seq

Wild-type (OreR) and Srrm234^eMIC−^ mutant flies were raised at 25°C and a 12-hour dark/12-hour light cycle. Late female and male L3 instar larvae and 24-hour female and male adults were collected separately, and their brains (>20 per sample) were dissected in 1× PBS and stored in RNAlater (QIAGEN, Venlo, The Netherlands). Total RNA was isolated using RNeasy Mini Kit (QIAGEN), and RNA quality was checked using Bioanalyzer (Agilent). A total of eight strand-specific Illumina libraries were prepared and sequenced at the CRG Genomics Unit. An average of 80 million 125-nt paired-end reads were generated for each sample.

### Visualization of genomics data

bigWig files with 3′ seq data of elav and fne mutants and bedGraph files with elav iCLIP data were downloaded from Gene Expression Omnibus (GEO; accession numbers GSE155534 and GSE146986, respectively). 3′ seq and CAGE data from different tissues were also downloaded from the GEO dataset GSE101603 and modENCODE (*5*). Data were visualized using Integrative Genomics Viewer web browser. For visualization of splice junctions (sashimi plots), we mapped reads using STAR and generated sashimi plots for specific genomic regions using ggsashimi (*72*).

### AS analysis of eMIC knockout brains

AS analysis was performed using PSI (percentage spliced in) values calculated with vast-tools v2.5.1 (*30*) for dm6 (VASTDB library: vastdb.dme.23.06.20.tar.gz), filtering out events with very low number of mapped reads (minimum quality score of LOW or higher). Global analysis of AS changes in eMIC mutant brains was done using a change in average PSI (ΔPSI) of 20 between mutant and control brains and requiring a minimum ΔPSI of 15 between genotypes for each sex independently, in either adult brains or third-instar larval central neural systems. Only AS events mapping to the coding sequence were considered (170 of 179 in the case of cassette exons).

For exon classification based on the regulation by the eMIC domain, we used the following criteria:

1) “eMIC-dependent”: cassette exons that have substantially lower inclusion in eMIC-mutant brains. We used the previous criteria ΔPSI ≤ −20 in either adult or larval samples and minimum ΔPSI ≤ −15 for each sex. We also added exons with a ΔPSI ≤ −10 in both sexes if the inclusion level in the knockout samples was very low (PSI ≤ 1).

2) “eMIC-sensitive”: exons not included in the above group that show a ΔPSI ≤ −10 in both sex-paired comparisons between wild-type and knockout samples, for either adult or larval samples. We also considered eMIC-sensitive exons that were enhanced by the overexpression of human SRRM4 in SL2 cells (*15*) with a ΔPSI ≥15 and that were not considered eMIC-dependent.

3) All other exons were considered eMIC-independent as long as they had sufficient coverage in our brain samples (minimum vast-tools score of LOW).

eMIC-dependent exons that were differentially spliced between males and females were defined as those with |ΔPSI| ≥ 20 between wild-type and eMIC-knockout brains in either sex and that have a |ΔPSI| ≥ 20 between male and female samples in control or mutant brains.

### Quantification of *Srrm234* last exon usage

We quantified alternative last exon usage at the *Srrm234* locus as the proportion of reads mapping to each of the three possible exon-exon junctions from the same common donor in the first eMIC-encoding exon to each possible acceptor site. The number of mapped reads for each of the three junctions was obtained from the eej2 output of vast-tools. The shared donor corresponded to donor 20, and the three quantified acceptors were 21, 22, and 23 from locus FBgn0035253.

### Genome-wide cell- and tissue type–specific splicing patterns

We collected publicly available RNA-seq data from fly adult tissues from different sources (table S1), quantified PSI values using vast-tools v2.5.1 (*30*), and grouped exons on the basis of their inclusion profiles. For this, we grouped tissues into eight groups: neural, sensory, muscle, digestive tract, salivary glands, ovary, testis, and sex glands. Next, to define tissue-enriched exons, we required a ΔPSI ≥25 between that tissue and the average of all other tissues and ΔPSI ≥15 between that tissue and the maximum PSI across all other tissues. Similar analyses but changing the direction of ΔPSI were done to define tissue-depleted exons. For the case of neural and sensory tissues, we excluded each other from the “all other tissues” group, given their partially overlapping cell-type composition. AS analysis across neural cell types was performed using a recently published dataset containing over 100 samples (*48*) together with other independent samples (see table S1 for full list of samples). We assessed global patterns of alternatively spliced exons by looking at exons with a PSI range ≥ 20 across neuronal and glial samples.

### Tissue- and cell type–specific profiles of eMIC-dependent exons

We classified eMIC-dependent exons into three groups based on their inclusion profiles across tissues: (i) “Shared”: exons that were not enriched in neural tissues with a PSI ≥ 40 in any non-neural tissue or with a ΔPSI ≤ 15 when comparing neural and non-neural samples, (ii) “Eye”: eye-enriched exons with a ΔPSI ≥ 20 between eye and other neural tissues (brain and thoracicoabdominal ganglion), and (iii) “Neural”: all other eMIC-dependent exons showing neural enrichment, as described in the previous section.

Analogously, we classified eMIC-dependent exons into five groups based on their inclusion profiles across neural cell types: (i) “Glia-shared”: exons with a PSI ≥ 50 in any glial sample, (ii) “PR-up”: exons with a ΔPSI ≥25 between photoreceptors and other neuronal types, (iii) “KC-down”: exons with a ΔPSI ≤ −25 between Kenyon cells and other neuronal types, (iv) “PR-down”: exons with a ΔPSI ≤ −25 between photoreceptors and other neuronal types, and (v) “pan-neuronal”: the rest of eMIC-dependent exons with neuronal enrichment.

To calculate sample-to-sample distances based on the inclusion profile of eMIC-dependent exons or all alternatively spliced exons across neural types, we used (1 − Pearson correlation) as the clustering distance between samples.

### AS analysis of mammalian exons

We quantified AS genome-wide using the same method as described above for *D. melanogaster*, i.e., vast-tools v2.5.1 for mouse mm10 (VASTDB library: vastdb.mm2.23.06.20.tar.gz) and human hg38 (VASTDB library: vastdb.hs2.23.06.20.tar.gz) genome assemblies. Definition of mouse eMIC-dependent and eMIC-sensitive exons was based on publicly available data of the double KD of *Srrm3* and *Srrm4* in N2A cells (*18*) and the knockout of *Srrm4* in mouse hippocampus and cerebral cortex (*12*). We classified exons as eMIC-dependent if they had an average ΔPSI ≤ −20 between mutant and control samples and minimum PSI range ≤ −15 between conditions or had an average ΔPSI ≤ −10 between mutant and control samples and maximum PSI ≤ 1 in mutant samples. eMIC-sensitive exons were those that were not included in the previous group and that had a ΔPSI ≤ −15 and PSI range ≤ −5 between *Srrm3/4* KD and control samples in N2A cells or that had a ΔPSI ≤ −10 between *Srrm4* knockout and control samples in both hippocampus and cortex. Exon groups used as controls: Neural, ASE, LowPSI, and HighPSI were defined as described in (*15*) (and see below) and are included in table S2. Definition of human eMIC-dependent exons was based on overexpression data of SRRM4 in HEK293 cells (*15*, *73*): We required a ΔPSI ≥ 40 between overexpression and control and |ΔPSI| ≤ 10 between replicates. Neural and ASE control groups were also taken from (*15*) and included in table S2.

### Analysis of exon/intron features

Analysis of the cis-regulatory code associated with eMIC-dependent splicing was done by comparing the eMIC-dependent and eMIC-sensitive exon sets to four control exon sets (table S2). We defined these group sets on their inclusion profiles across the eight tissue groups defined above: (i) “HighPSI”: highly included exons with a minimum PSI > 90 across all tissues with sufficient read coverage (vast-tools score LOW or higher), (ii) “LowPSI”: lowly included exons with a maximum PSI < 10 across all tissues with sufficient read coverage, (iii) “Neural”: neural-enriched exons (as defined above) that are not regulated by the eMIC domain, and (iv) “ASE”: alternatively spliced exons with sufficient read coverage in at least in three different tissues and that are alternative (10 ≤ PSI ≤ 90) in at least 25% of all samples. HighPSI exons were down-sampled to 1000 exons by random selection. We only considered exons mapping to coding sequences (as for eMIC-regulated exons) to avoid biases due to the differential location of exons within transcripts.

We calculated the strength of the donor and acceptor splice sites (5′ ss and 3′ ss/AG, respectively) according to maximum entropy score models. To test for differences in the median of the scores for each feature, we used Mann-Whitney *U* tests. To generate the RNA maps in Fig. 6 and fig. S6, we used rna_maps function from Matt v1.3 (*74*) using sliding windows of 27 nt. Polypyrimidine tracts were searched as YYYY tetramers, CU-rich motifs included the motifs UCUC and CUCU, and the *D. melanogaster* branch point consensus sequence used was CUAAY. To estimate false discovery rate (FDR) values in the RNA maps, we used a permutation test using 1000 permutations and a threshold of FDR < 0.05 as implemented in Matt. RIME was calculated for the skipping isoform of each exon. The total length of the intron was calculated as the sum of the alternative exon and the upstream and downstream introns, and mean exon length was calculated for the adjacent constitutive exons.

### Protein impact prediction

Exons detected by vast-tools that mapped to coding sequences were classified following the description in (*11*), as provided in VastDB (version 2.2 of protein predictions). Briefly, exons were predicted to disrupt the coding sequence if their inclusion or skipping would induce a frameshift in their open reading frame or if they would induce a premature stop codon predicted to be targeted by nonsense-mediated decay (NMD) or to truncate the protein by more than 300 amino acids or more than 20% of the reference isoform. The rest of coding sequence–mapping exons are predicted to preserve the transcript coding potential.

### AS analysis of RBP perturbations

Published data of CNSs from *elav* and *fne* mutant first-instar larvae were analyzed using vast-tools v2.5.1. We considered “elav/fne-dependent” exons those with an average ΔPSI ≤ −20 and a PSI range ≤ −15 between elav/fne double-knockout and control samples. We analyzed the effect of knocking down a collection of RBPs in *Drosophila* SL2 cells using data from modENCODE and REF-U2af. We processed these data with vast-tools and quantified the effect of each KD on different types of exons: eMIC-dependent, eMIC-sensitive, Neural, and other alternatively spliced exons (ASEs, defined above). We tested the difference in the ΔPSI of each KD on eMIC-dependent exons and compared it to the effect on ASEs. We compared the differences between the two exon groups for each RBP using Mann-Whitney *U* tests, correcting for multiple testing with the Benjamini-Hochberg method.

To test the overlap with other splicing programs controlled by different RBPs, we used a publicly available dataset on RBP KDs in the fly brain (table S1) (*40*). We calculated the ΔPSI between each RBP KD and the control green fluorescent protein–KD sample for all eMIC-dependent exons and considered as regulated those with an |ΔPSI| ≥ 20 or 25. For this and the SL2 dataset, we assessed the overall KD efficiency of each RBP by calculating the fold change in gene expression for each sample (fig. S8, B and D).

The same approach was followed to calculate the effect of knocking down the human U2af38 and heph orthologs in HEK293 cells: U2AF1 and PTBP1/2, respectively, and for mouse Ptbp1/2 KD in embryonic stem cells. All data sources are included in table S1, and necessary files to run vast-tools are indicated in the “Data and materials availability” section.

### Differential gene expression analysis

Gene expression quantification was done using vast-tools v2.5.1. For analyses of eMIC mutant brains, we considered female and male samples as replicates given their high degree of similarity. To quantify differential gene expression (DGE), raw gene counts calculated with vast-tools for wild-type and mutant adult brains were used as input for edgeR. DGE was estimated using the likelihood ratio test between genotypes taking the sex factor into consideration (~genotype + sex). FDRs were calculated using the Benjamini-Hochberg method. DEGs with an FDR ≤ 0.1 and fold change ≥1.5 in adult mutant brains are listed in table S4. Statistical assessment of the number of activity-regulated genes that are differentially expressed in eMIC mutant brains was determined using Fisher’s exact test.

### Analysis of neuronal-activity DGE and AS

We assessed the effect of sustained neuronal activation on gene expression and AS using a publicly available dataset using two stimulation paradigms: KCl-induced depolarization and optogenic activation (*52*). We assessed DGE using the “exact test” method as implemented by edgeR, comparing each time point with its corresponding unstimulated sample for each of the two stimulation paradigms. FDR values were calculated using the Benjamini-Hochberg method. We considered activity-regulated genes with an FDR value lower than 5% and with a fold change ≥1.5 compared with control samples at any time point. PSI values for all exons mapping to coding sequences genome-wide were calculated as described above.

### Gene group classification of genes with alternatively spliced exons

We classified genes harboring alternatively spliced exons into functional groups according to the classification directly downloaded from FlyBase gene groups (“gene_group_data_fb_2020_01.tsv”). This classification is hierarchical, so we looked at two levels: the “bottom” categories with very specific gene subgroups and the “top” level group for each subgroup. We did this analysis for three types of exons: eMIC-dependent, neural non–eMIC-regulated, and other alternatively spliced exons. We plotted both the number of genes per group and their proportion of the total number of group members.

### Sequence conservation of the genomic region surrounding alternatively spliced exons

phastCons data files were downloaded from UCSC Genome Browser server. For mouse, we used phastCons data from an alignment of 60 vertebrate genomes, and for *D. melanogaster*, we used data from the alignment of 27 insect species. We averaged phastCons scores with respect to the start and end of our six previously defined exon groups for both species. We removed overlapping gene elements that may distort the signal such as upstream and downstream exonic sequences if these are closer than 150 bp and the first 10 nt of the upstream intron and the last 30 nt of the downstream intron to avoid signals coming from the splicing donor and acceptor sites.

### Exon-level conservation with liftOver

To look at genomic conservation of exons, we used UCSC liftOver. For *D. melanogaster*, we used liftOver chain files for dm6 genome annotation with all available species. For mouse, we used representative species covering a similar time window as the available species for *Drosophila*, i.e., rat, human, cow, opossum, chicken, and frog. We defined four levels of exon conservation: (i) The entire region is missing from the chain file in the second species (including adjacent constitutive exons). (ii) Adjacent exons can be liftOvered but not the alternatively spliced exon region. (iii) The genomic region encompassing the alternatively spliced exon can be liftOvered, but none of the exon splice sites are present. (iv) At least one of the splice sites from the alternatively spliced exon is present (either the acceptor or the donor sites). We considered exons to be conserved at the genome level if they belong to the last group.

### Overlap of eMIC-dependent splicing programs between fly and mouse

We defined orthology relationships between mouse and fly using DIOPT (www.flyrnai.org/diopt). For conservation analysis of eMIC-dependent exons, we first assigned gene orthology requiring a DIOPT score ≥ 2 and “best score” in at least one direction. Orthologous genes with eMIC-dependent exons in both species were then pairwise aligned using MUSCLE. Exons were considered orthologous if they are in the same intron in both species, i.e., having the same adjacent exons.

### GO analysis

To avoid biases derived from species-specific gene annotations, we followed a strategy based on orthology relationships to assess GO enrichment. First, we generated two lists of genes for each species: eMIC-dependent exon-containing genes and a background gene set. The background list contained those genes with a minimum expression level similar to eMIC-containing genes in the datasets used for calling of eMIC dependency: cRPKM ≥5 in adult fly brains or cRPKM ≥3 in N2A cells. These gene lists were then transformed to their human orthologs with similar criteria as specified above: DIOPT score ≥ 2 and best score required (“best-reverse” score not sufficient). We then run GO enrichment analysis using GOrilla (http://cbl-gorilla.cs.technion.ac.il/) with the “two unranked lists of genes” module using the human annotation. We joined the output obtained using the human orthologs of mouse and fly genes for each category (Process, Function, and Component) and visualized them using ggplot2. Similar results were obtained when swapping the human ortholog background lists generated using either the fly or mouse data. Last, we also performed the same analysis using the fly orthologs of mouse genes and running GOrilla using the *D. melanogaster* GO annotation to explore GO terms associated with fly-specific biological processes. All GO terms enriched for the Function and Component categories are represented in Fig. 9C and fig. S10, and all enriched GO terms are listed in table S3.

## Supplementary Materials

### This PDF file includes:

Figs. S1 to S12 Legends for tables S1 to S5 Legends for movies S1 to S3 References

## Notes

View/request a protocol for this paper from *Bio-protocol*.
