# Supplementary material for: Parallel evolution of a splicing program controlling neuronal excitability in flies and mammals
Source: Sci Adv. 2022 Jan 28;8(4):eabk0445. doi: 10.1126/sciadv.abk0445 (PMC8797185; doi:10.1126/sciadv.abk0445)
Supplement: Supplementary file 1 — Figs. S1 to S12 Legends for tables S1 to S5 Legends for movies S1 to S3 References [file sciadv.abk0445_sm.pdf]

Science Advances
AAAS

Supplementary Materials for

Parallel evolution of a splicing program controlling neuronal excitability in flies and mammals

Antonio Torres-Méndez*, Sinziana Pop, Sophie Bonnal, Isabel Almudi, Alida Avola, Ruairí J. V. Roberts, Chiara Paolantoni, Ana Alcaina-Caro, Ane Martín-Anduaga, Irmgard U. Haussmann, Violeta Morin, Fernando Casares, Matthias Soller, Sebastian Kadener, Jean-Yves Roignant, Lucia Prieto-Godino*, Manuel Irimia*

*Corresponding author. Email: mirimia@gmail.com (M.I.); lucia.prietogodino@crick.ac.uk (L.P.-G.); antonio.torres@crg.eu (A.T.-M.)

Published 28 January 2022, *Sci. Adv.* **8**, eabk0445 (2022)
DOI: 10.1126/sciadv.abk0445

The PDF file includes:

Figs. S1 to S12
Legends for tables S1 to S5
Legends for movies S1 to S3
References

Other Supplementary Material for this manuscript includes the following:

Tables S1 to S5
Movies S1 to S3

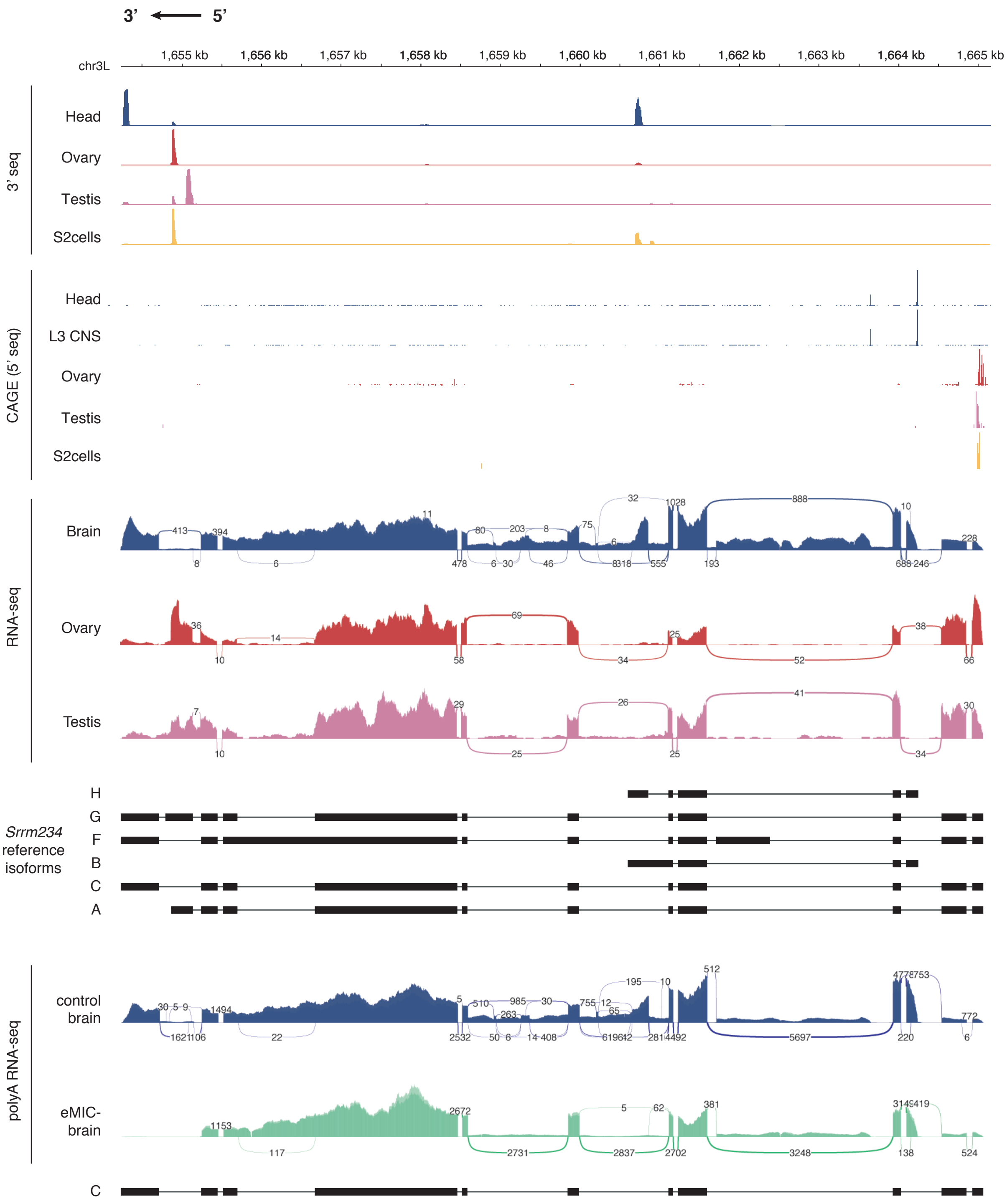

Supplementary Figure 1. *Srrm234* alternative isoforms across tissues

Transcriptomic data mapping at the *Srrm234* (CG7971) gene. From top to bottom: 3′ seq data from (*77*); CAGE-seq (cap analysis gene expression) data from (*5*); RNA-seq data from Fly Atlas 2 (*19*); main transcript isoforms annotated in FlyBase; and RNA-seq data generated in this study for control and eMIC- adult brains. Numbers of reads mapping to each exon-exon junction are indicated.

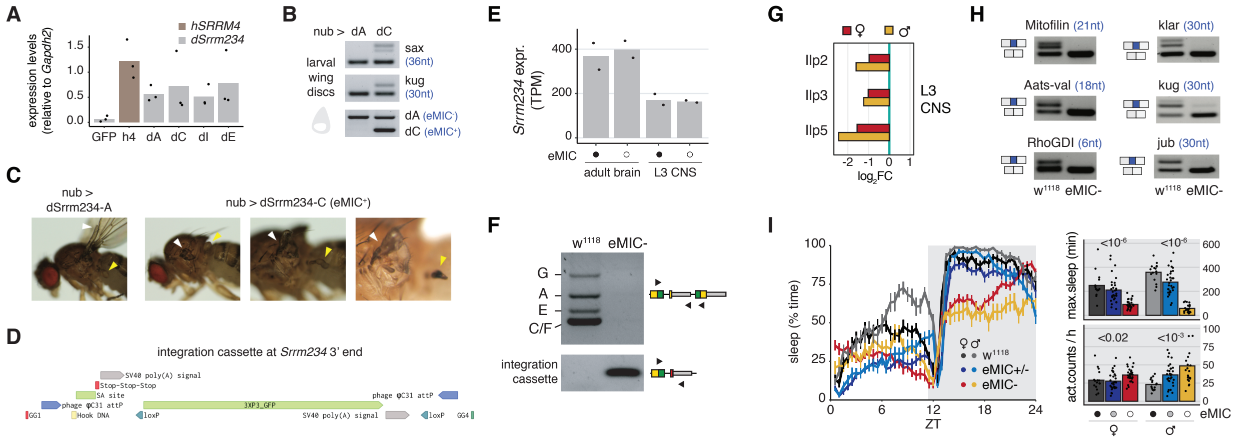

Supplementary Figure 2. Phenotypic characterization of flies lacking the eMIC domain

(A) Expression levels as quantified by qPCR of heterologous expressed *Srrm234* constructs (*Drosophila* isoforms A, C, I, E, and human *SRRM4*) in SL2 cells, relative to the housekeeping *Gapdh2* gene.

(B) RT-PCR assays of short alternatively spliced exons and *Srrm234* 3′ end region, from larval wing discs overexpressing *dSrrm234* isoform A or C (dA, dC) under a nubbin (nub-GAL4) driver line.

(C) Representative wings (white arrows) and halteres (yellow arrows) of flies expressing *Srrm234*-derived transgenes under the control of nub-GAL4 (*nubbin*) driver lines. eMIC expression results in absence of wings and bigger, elongated or necrotic halteres (from left to right).

(D) Integration cassette introduced at the 3′ end of *Srrm234* replacing the endogenous sequence that encodes for the eMIC domain (region delimited by guide RNAs in Figure 2A).

(E) Expression levels of *Srrm234* as quantified from RNA-seq, in transcripts per million (TPM). Black circles: eMIC+/+ control samples, empty circles: eMIC- samples.

(F) RT-PCRs from fly heads of the 3′ end region of S*rrm234* either for the endogenous allele (top gel) or for the eMIC- allele (bottom).

(G) Change in expression levels of neuronally secreted insulin-like peptides (Ilps) in eMIC- L3 CNSs relative to control CNSs, as quantified from RNA-seq data.

(H) RT-PCRs of alternatively spliced exons in fly heads for control (w^{1118}) and eMIC- flies. In brackets, exon lengths.

(I) Sleep patterns of 21-day-old flies in 12h light – 12h dark cycles. Left, average time flies spend sleeping (inactive for ≥ 5min) at different times of the light:dark cycle. ZT: Zeitgeber Time (switch from light to dark conditions), vertical lines: standard error of the mean. Top right, maximum sleep episode during the night. Bottom right, total number of activity counts per hour during the night. P-values from Mann-Whitney U tests comparing with w^{1118} controls.

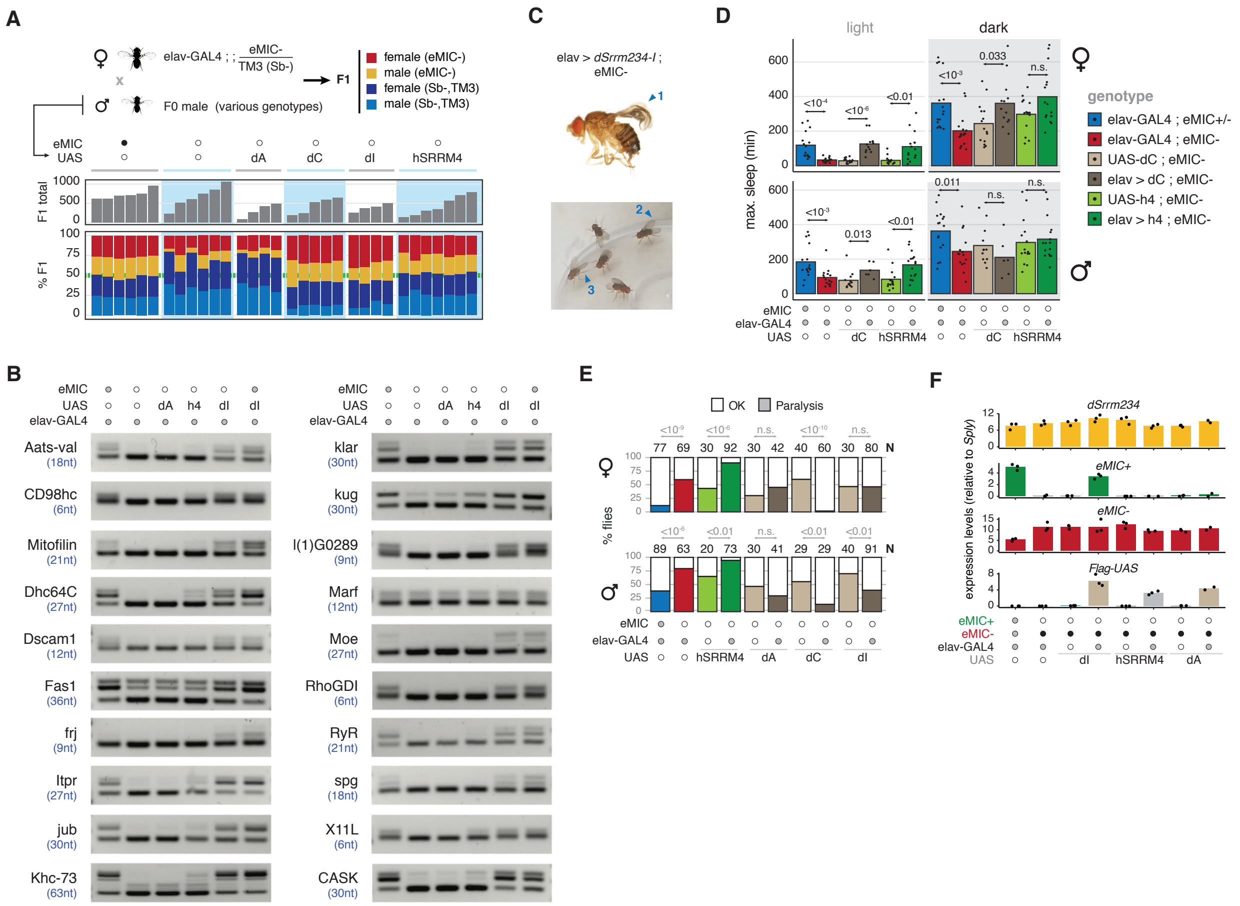

Supplementary Figure 3. Phenotypic characterization of flies overexpressing *Srrm234* constructs in the eMIC- background

(A) Top, mating scheme for the determination of relative fitness values presented in Figure 3B. Sb: *stubble*. Middle, total number of flies quantified per experiment. Bottom, allele frequencies in the F1 generation.
(B) RT-PCR assays on female fly heads of candidate eMIC-regulated exons. dI: *Drosophila* Srrm234-I, h4: human SRRM4, dA: *Drosophila* Srrm234-A.
(C) Deleterious effects of overexpressing dSrrm234-I pan-neuronally in the eMIC- background: failed wing expansion (1) and open positioning of wings (2) and legs (3). Photo credit: Antonio Torres-Méndez.
(D) Maximum duration of sleep episodes during light or dark conditions (12h-12h) for each sex. P-values from Mann-Whitney U-tests, n.s.: $p > 0.05$.
(E) Sensitivity of adult flies to mechanical stimulation (10s vortex) for each sex. On top of each graph: number of flies tested and p-values from Fisher's exact tests.
(F) Expression levels as quantified by qPCR assays of *Srrm234*, each of the 3′ end alleles (eMIC+ and eMIC-), and UAS-transgenes in female fly heads, relative to the housekeeping *Sply* gene.

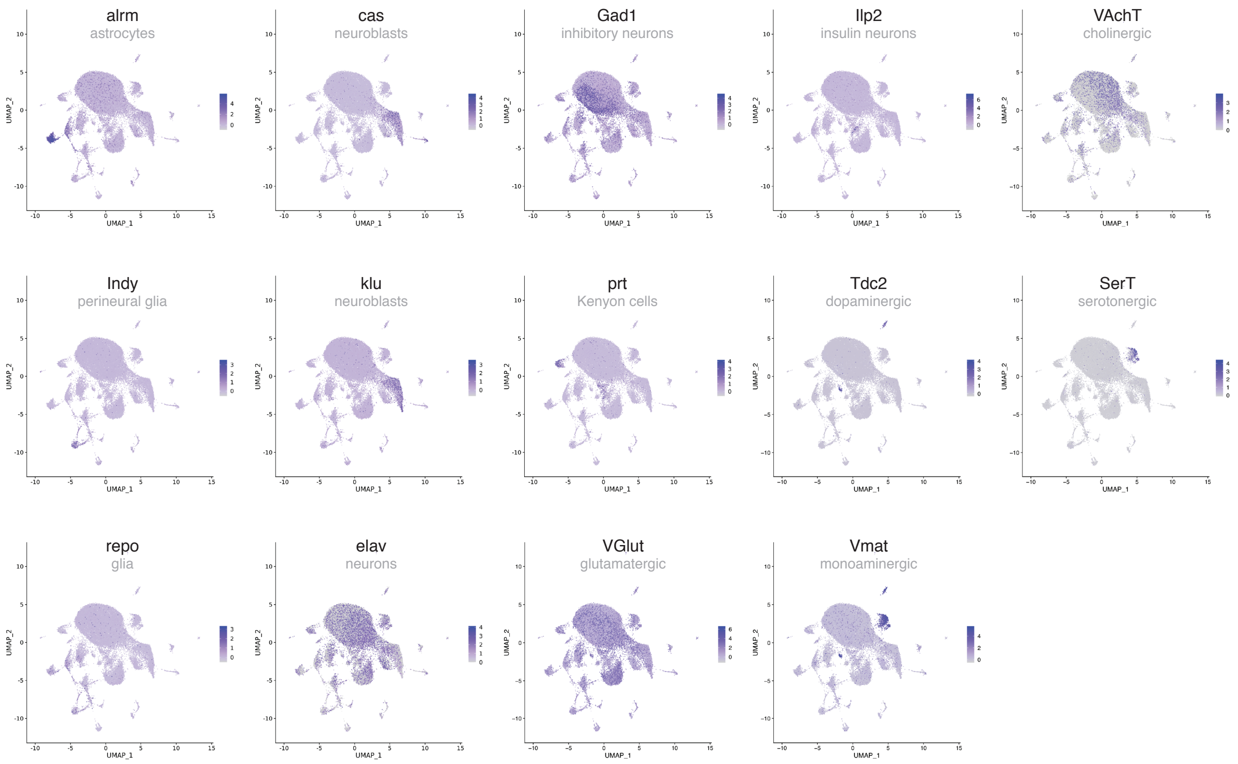

Supplementary Figure 4. Cell-type markers in single cell RNA-seq data

Expression of different genes used as markers to define neuronal and glial populations in the larval nervous system highlighted in Figure 5B.

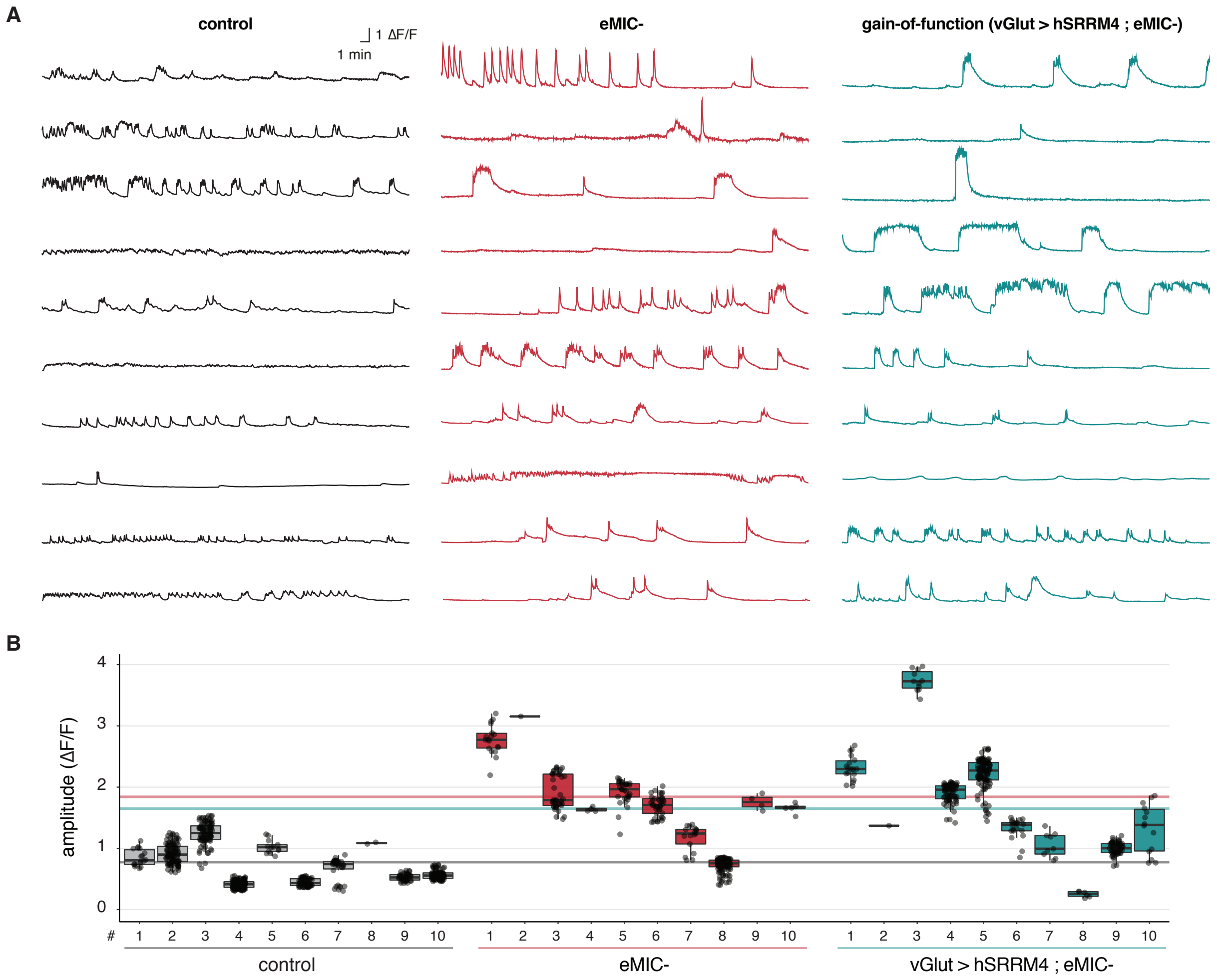

Supplementary Figure 5. Fictive locomotion experiments in L3 larvae

(A) Traces representing the mean activity across all segments of the ventral nerve cord (VNC) for each sample. Activity is calculated based on the change in fluorescence (F) of the GCaMP7b calcium indicator over the base line (ΔF/F).

(B) Distribution of amplitudes for every peak of activity in each sample. Horizontal lines mark the average values for each genotype.

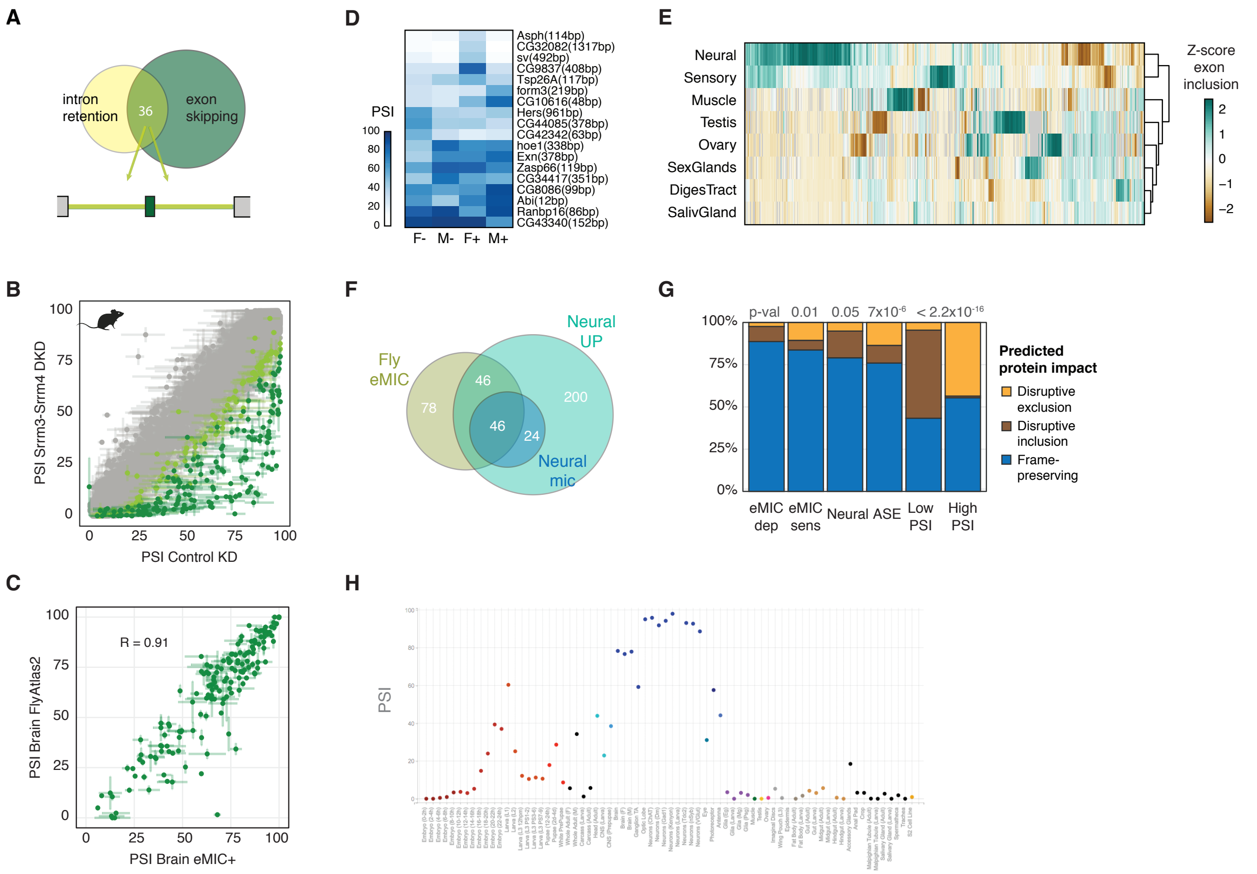

Supplementary Figure 6. Regulation of alternative splicing by the eMIC domain

(A) Overlap of alternatively retained introns and alternatively spliced exons (introns adjacent to identified alternatively spliced exons) affected in eMIC- brains.
(B) PSI of mouse eMIC-dependent exons upon perturbation of *Srrm3* and *Srrm4* mRNA levels. Data from (*12*, *18*). KD: knock-down, DKD: double knock-down. Error bars indicate the PSI range across replicates.
(C) PSI values of eMIC-dependent exons in our controls (x-axis) and wild-type brain samples from FlyAtlas 2 (*19*) (y-axis). Error bar ends indicate the difference between male and female samples.
(D) Inclusion levels of exons alternatively spliced between sexes in eMIC- and control adult brain samples as quantified by RNA-seq. F: female, M: male, +: eMIC+, -: eMIC-. In grey: insufficient read coverage.
(E) Tissue-specific alternative exons in *Drosophila*. Data sources included in Table S1.
(F) Overlap of eMIC-dependent exons and neural-enriched exons. Within neural exons, microexons (mic, exons shorter than 28 nt) are highlighted in dark-blue.
(G) Prediction of the effect of alternatively spliced exons on their cognate proteins. dep: dependent, sens: sensitive, ASE: other AS exons, PSI: percent spliced in, Low PSI: cryptic exons, High PSI: constitutive exons.
(H) Representative example of the PSI quantification across tissues for AS events available at vastdb.crg.eu.

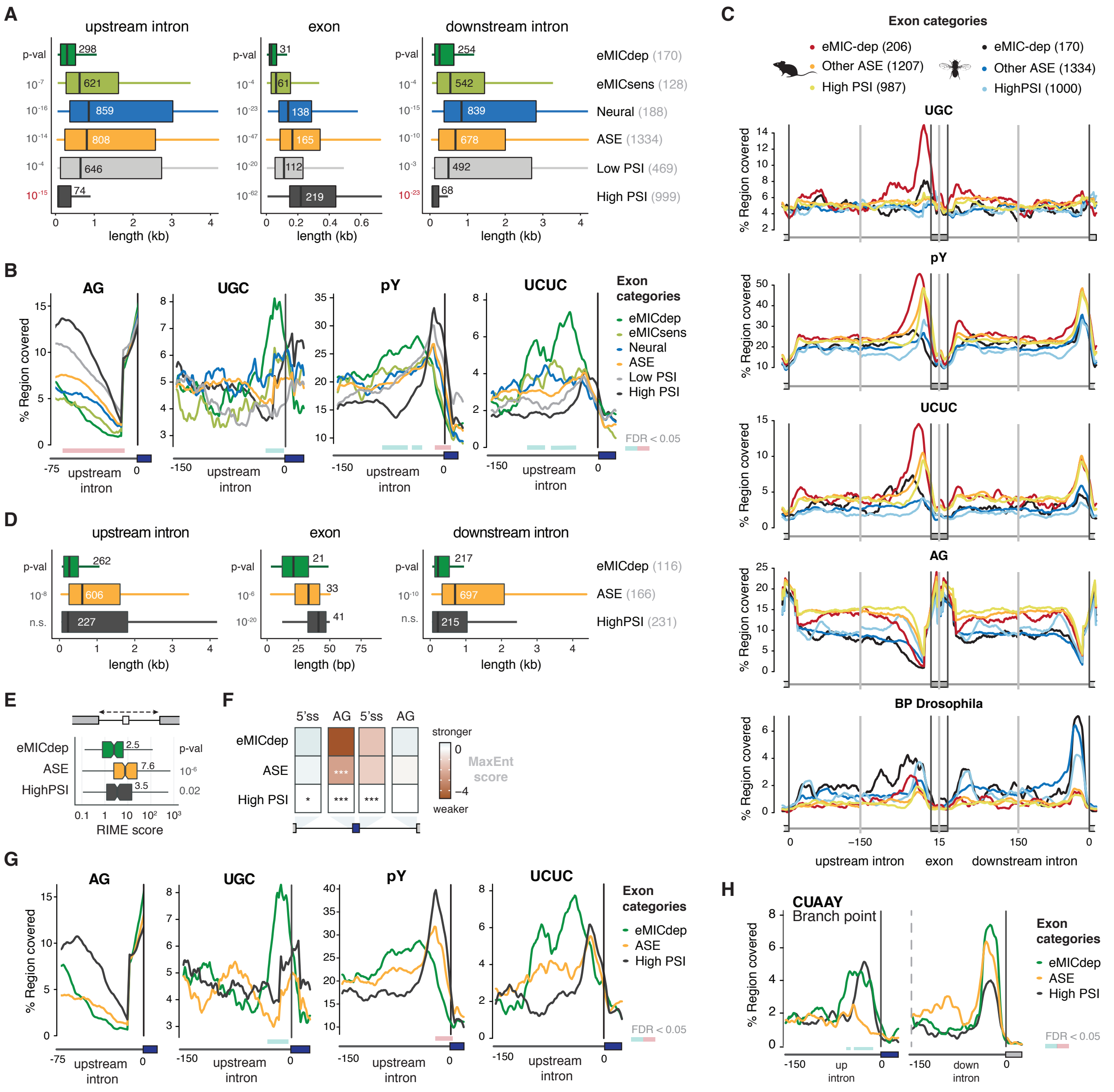

Supplementary Figure 7. *cis*-regulatory features associated with eMIC splicing in mice and flies

(A) Length of the exon and neighbouring introns for six exon groups, from top to bottom: eMIC-dependent, eMIC-sensitive, Neural, Other AS exons (ASE), cryptic and constitutive exons (Table S2). Box limits represent interquartile ranges; central lines, median values (also indicated with numbers). P-values from Mann-Whitney U tests are shown for the comparison of each class against eMIC-dependent exons. Red font indicates that the difference goes in the opposite direction. Number of exons per group is indicated in parentheses.

(B) RNA maps for motifs enriched or depleted in the intronic region upstream of eMIC-dependent exons. From left to right: distribution of AG motifs in the 75 nt upstream of the alternative exon start (3′ ss), UGC motif distribution close to the 3′ ss, Polypyrimidine tract profiles for YYYY tetramers (pY) or CU-rich (UCUC/CUCU) tetramers. Length of sliding

window: 15 nt for AG and UGC and 27 nt for the others. Regions with a significant difference in the motif coverage (FDR < 0.05) compared to ASE group are marked with a coloured rectangle underneath.

(C) RNA-maps of motifs enriched or depleted in the intronic regions flanking eMIC-dependent exons in mouse and *Drosophila*. From top to bottom: UGC motifs, polypyrimidine tetramers, alternating CU-rich tetramers, AG dinucleotide and the consensus branch-point sequence in *Drosophila* CUAAY. Length of sliding window: 27 nt.

(D-E) Length of the exon and neighbouring introns for exons shorter than 51 bp of the groups in panel A (D) and Ratio of Intron to Mean Exon length (RIME) score for introns harbouring those exons (G). Box limits represent interquartile ranges; central lines, median values (also indicated with numbers). P-values from Mann-Whitney U tests are shown, n.s.: p > 0.05. Number of exons per group is indicated in parentheses.

(F) Maximum entropy scores for the 5′ splice site and AG region, relative to constitutive (High PSI) short exons. P-values from Mann-Whitney U tests are shown for the comparison with eMIC-dependent exons (shorter than 51 bp).

(G-H) RNA maps for motifs enriched or depleted in the intronic region upstream of eMIC-dependent microexons. (G) From left to right: distribution of AG motifs in the 75 nt upstream of the alternative exon start (3′ ss), UGC motif distribution close to the 3′ ss, Polypyrimidine tract profiles for YYYY tetramers (pY) or CU-rich (UCUC/CUCU) tetramers, (H) *Drosophila* branch point consensus sequence (CUAAY). Length of sliding window: 27 nt. Regions with a significant difference in the motif coverage (FDR < 0.05) compared to ASE group are marked with a coloured rectangle underneath.

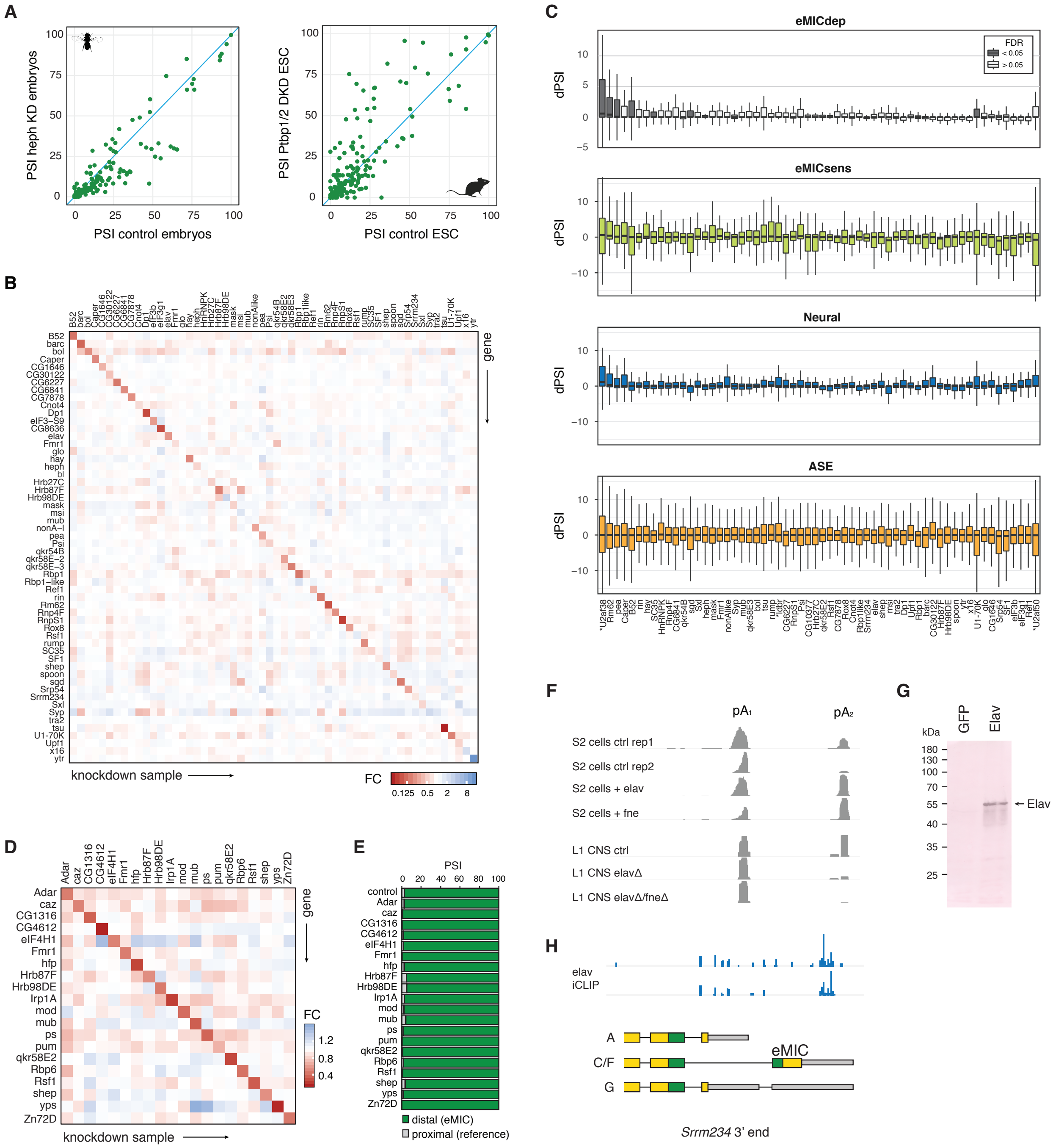

Supplementary Figure 8. Cross-talk between eMIC splicing and other RBPs

(A) Inclusion of eMIC-dependent exons in PSI (percent spliced in) upon perturbation of *PTB* expression levels. Left, *heph* (*Ptbp1/2/3 Drosophila* ortholog) knockdown (KD) and control embryos from (*33*). Right, *Ptbp1/2* double knockdown (DKD) and control mouse embryonic stem cells (ESC) from (*78*).

(B,C) Expression of RNA binding proteins (B) and change in inclusion levels (dPSI) for different types of exons upon knockdown of an array of RBPs in *Drosophila* SL2 cells (C). Data from modENCODE (*34*) or (*36*) (the latter are marked with an asterisk). FC: fold-change relative

to control KD samples, eMICdep: eMIC-dependent exons, eMICsens: eMIC-sensitive exons, ASE: other alternatively spliced exons. In boxplots, centre of the box marks median values, box limits mark interquartile ranges (IQR) and whiskers 1.5 IQR.

(D,E) Expression of RBPs (D) and AS at the 3′ end of *Srrm234* (E) upon KD of several RBPs in *Drosophila* adult brains. Data from (*40*).

(F) 3′ seq reads at the *Srrm234* locus upon perturbation of *elav/fne* levels: overexpression in SL2 cells or knockout in L1 larva central neural system (CNS). pA: poly-adenylation site, ctrl: control, rep: replicate. Data from (*41*).

(G) Elav expression in SL2 cells detected by western blot (related to Figure 7F). Ponceau staining was used as loading control.

(H) Elav iCLIP tags at the *Srrm234* 3'end region from adult heads. Data from (*42*). Bottom, annotated *Srrm234* isoforms based on AS and poly-adenylation at this region.

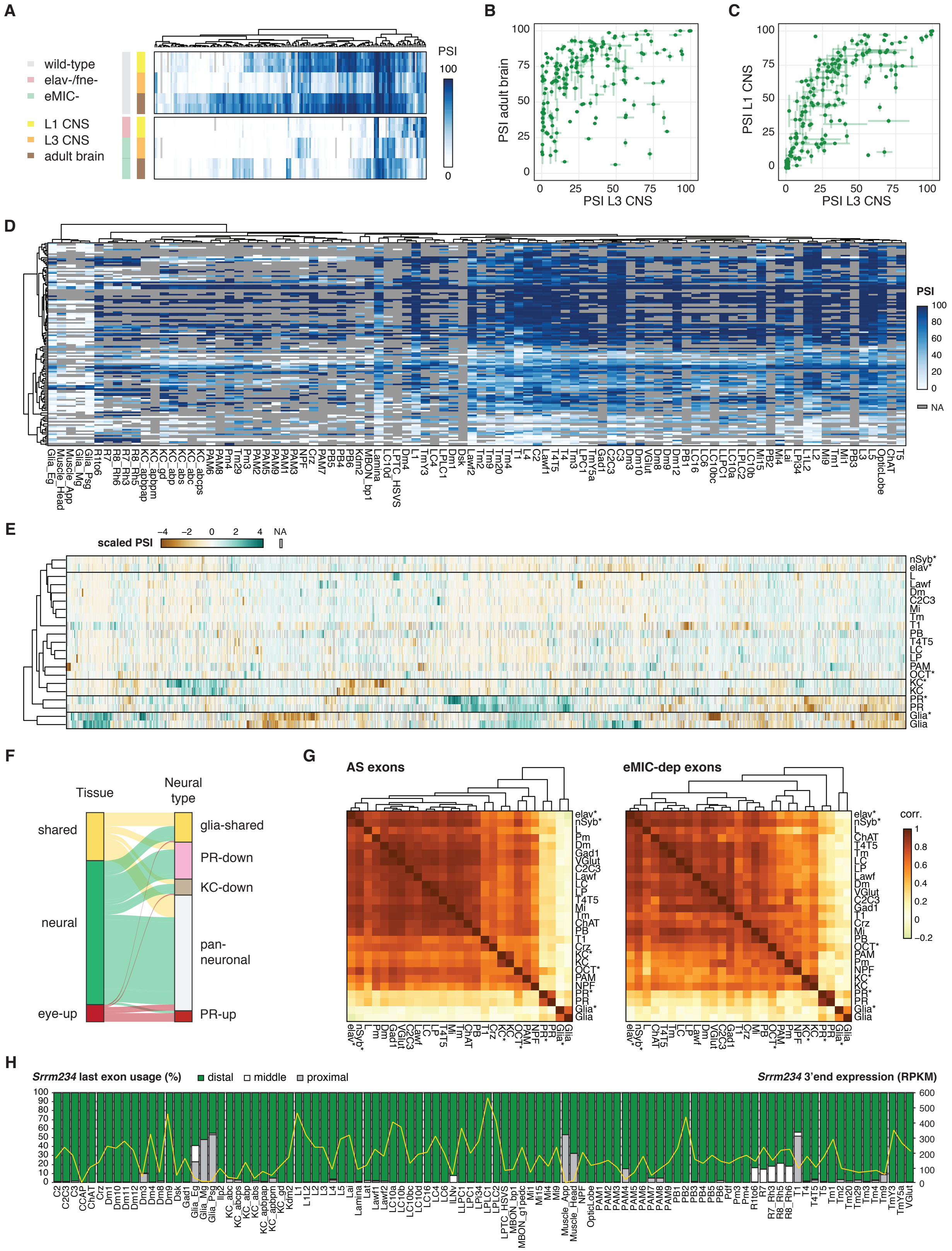
A
wild-type
elav-/fne-
eMIC-
L1 CNS
L3 CNS
adult brain
PSI
100
0
B
PSI adult brain
PSI L3 CNS
C
PSI L1 CNS
PSI L3 CNS
D
PSI
NA
E
scaled PSI
NA
nSyb*
elav*
L
Lawf
Dm
C2C3
Mi
Tm
T1
PB
T4T5
LC
LP
PAM
OCT*
KC*
KC
PR*
PR
Glia*
Glia
F
Tissue
Neural type
shared
neural
eye-up
glia-shared
PR-down
KC-down
pan-neuronal
PR-up
G
AS exons
eMIC-dep exons
corr.
H
Srrm234 last exon usage (%)
distal
middle
proximal
Srrm234 3'end expression (RPKM)

Supplementary Figure 9. Inclusion of eMIC-dependent exons across neural cell types

(A) Heatmap for the inclusion levels of eMIC-dependent exons in L1 and L3 central neural system (CNS) and adult brains for control and mutant flies. PSI: Percent Spliced In.
(B) Comparison of the PSI values of eMIC-dependent exons in larval CNS and adult brains. Error bar ends indicate the difference between male and female samples. Data generated in this study.
(C) PSI values of eMIC exons in L1 and L3 larva CNS. L1 data from (*41*), error bars representing the PSI range across replicates. L3 data, from FlyAtlas 2 and this study, error bar ends indicating PSI values in each source.
(D) eMIC-dependent exon inclusion across cell types in the *Drosophila* adult optic lobe. Data from (*48*). PSI: percent spliced in. NC: no sufficient read coverage.
(E) Inclusion levels (scaled for each exon) of all alternatively spliced (AS) exons among different cell types in the fly optic lobe. Sample sources are listed in Table S1.
(F) Alluvial plot depicting the overlap between groups of eMIC-dependent exons based on their inclusion profile across tissues (left) or neural cell types (right).
(G) Sample to sample correlation (corr.) distance matrix for different cell types in the fly optic lobe using either all AS exons (left) or eMIC-dependent exons only (right). PR: photoreceptors, KC: Kenyon cells, OCT: octopaminergic neurons, nSyb: all neurons.
(H) Alternative last exon usage and gene expression levels of *Srrm234* (grey line) across cell types in the optic lobe. Data from (*48*). RPKM: corrected reads per kilobase per million mapped reads.

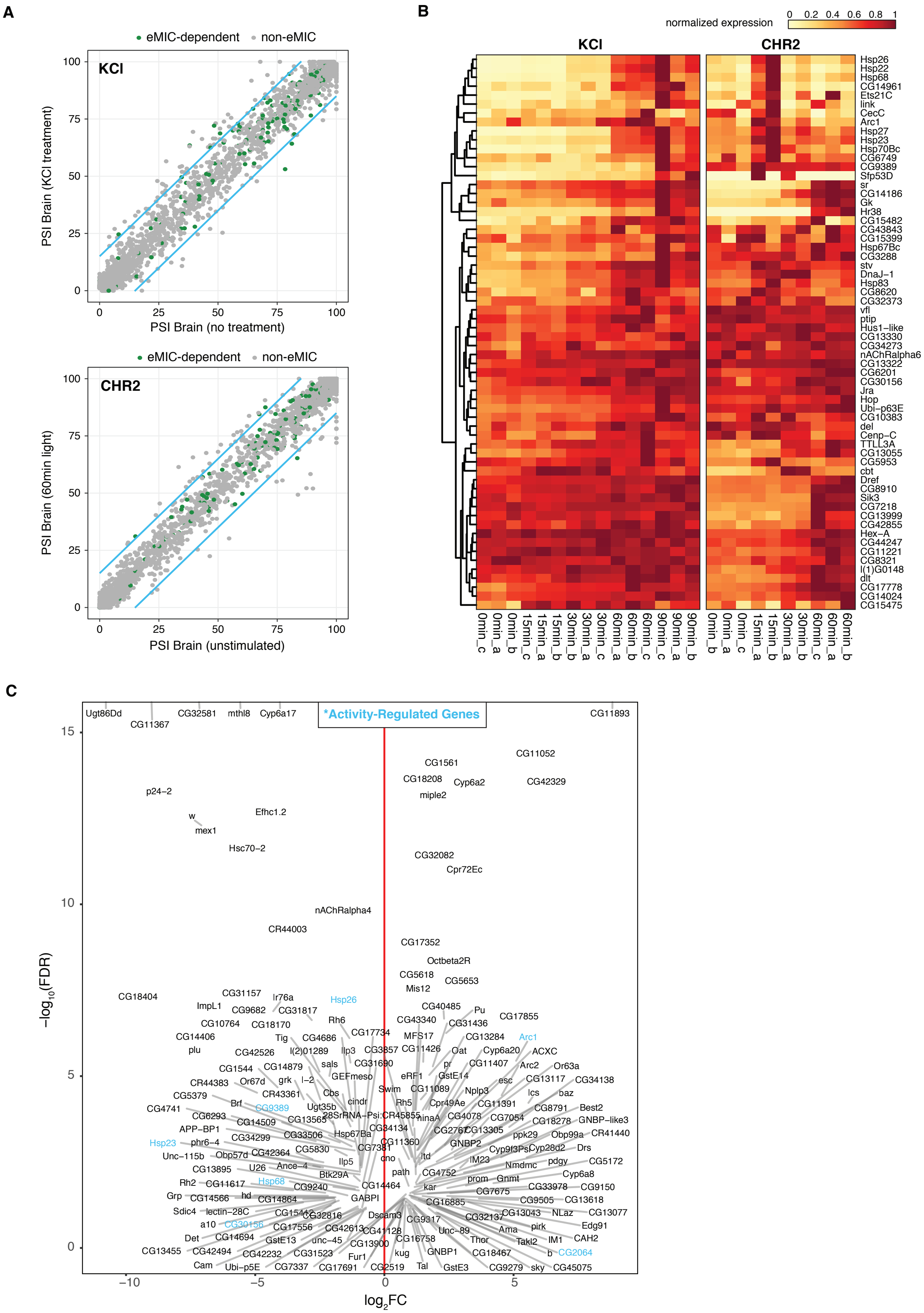
A
eMIC-dependent
non-eMIC
KCl
PSI Brain (KCl treatment)
PSI Brain (no treatment)
CHR2
PSI Brain (60min light)
PSI Brain (unstimulated)
0
25
50
75
100
B
normalized expression
0 0.2 0.4 0.6 0.8 1
KCl
CHR2
Hsp26
Hsp22
Hsp68
CG14961
Ets21C
link
CecC
Arc1
Hsp27
Hsp23
Hsp70Bc
CG6749
CG9389
Sfp53D
sr
CG14186
Gk
Hr38
CG15482
CG43843
CG15399
Hsp67Bc
CG3288
stv
DnaJ-1
Hsp83
CG8620
CG32373
vfl
ptip
Hus1-like
CG13330
CG34273
nAChRalpha6
CG13322
CG6201
CG30156
Jra
Hop
Ubi-p63E
CG10383
del
Cenp-C
TTLL3A
CG13055
CG5953
cbt
Dref
CG8910
Sik3
CG7218
CG13999
CG42855
Hex-A
CG44247
CG11221
CG8321
l(1)G0148
dlt
CG17778
CG14024
CG15475
0min_c
0min_a
0min_b
15min_c
15min_a
15min_b
30min_b
30min_a
30min_c
60min_a
60min_b
60min_c
90min_c
90min_a
90min_b
0min_b
0min_a
0min_c
15min_c
15min_b
15min_a
30min_a
30min_b
60min_c
60min_a
60min_b
C
*Activity-Regulated Genes
−log10(FDR)
log2FC
Ugt86Dd
CG11367
CG32581
mthl8
Cyp6a17
CG11893
CG1561
CG11052
CG18208
Cyp6a2
CG42329
miple2
p24-2
Efhc1.2
w
mex1
Hsc70-2
CG32082
Cpr72Ec
nAChRalpha4
CR44003
CG17352
Octbeta2R
CG5618
CG5653
Mis12
CG18404
CG31157
Ir76a
Hsp26
ImpL1
CG9682
CG31817
CG40485
Pu
CG17855
Rh6
CG10764
CG18170
CG43340
CG31436
CG14406
Tig
CG4686
CG17734
MFS17
CG13284
Arc1
plu
CG42526
l(2)01289
Ilp3
CG3857
CG11426
Oat
Cyp6a20
ACXC
CG1544
CG14879
sals
CG31690
pr
CG11407
Arc2
Or63a
CR44383
Or67d
grk
l-2
GEFmeso
eRF1
GstE14
esc
CG13117
CG34138
CG5379
CR43361
Cbs
Swim
CG11089
Nplp3
Ics
baz
CG4741
Brf
CG9389
Ugt35b
cindr
Rh5
Cpr49Ae
CG11391
CG8791
Best2
CG6293
CG14509
CG13565
28SrRNA-Psi:CR45855
ninaA
CG4078
CG7054
CG18278
GNBP-like3
APP-BP1
CG34299
CG33506
Hsp67Ba
CG34134
CG2767
CG13305
ppk29
Obp99a
CR41440
Hsp23
phr6-4
CG11360
GNBP2
Cyp9f3Psi
Cyp28d2
Drs
Unc-115b
Obp57d
CG42364
CG5830
CG7381
ltd
IM23
pdgy
CG5172
CG13895
U26
Ance-4
Ilp5
cno
Nmdmc
Cyp6a8
Rh2
CG11617
Hsp68
Btk29A
path
CG4752
prom
Gnmt
CG33978
CG9150
Grp
CG14566
hd
CG14864
CG9240
CG14464
kar
CG7675
CG9505
CG13618
Sdic4
lectin-28C
CG15414
CG32816
GABPI
CG16885
CG13043
NLaz
CG13077
a10
CG30156
CG17556
CG42613
Dscam3
CG9317
CG32137
pirk
Edg91
Det
CG14694
GstE13
unc-45
CG41128
Unc-89
Ama
IM1
CAH2
CG13455
CG42494
CG42232
CG31523
CG13900
CG16758
Thor
Takl2
b
CG2064
Cam
Ubi-p5E
CG7337
CG17691
Fur1
kug
GNBP1
CG18467
CG2519
Tal
GstE3
CG9279
sky
CG45075
15
10
5
0
−10
−5
0
5

Supplementary Figure 10. Neuronal-activity-regulated transcriptomic changes and differential gene expression in eMIC- brains

(A) PSI of all exons with sufficient read coverage upon two stimulation paradigms: KCl-induced depolarization (top), and pan-neuronal optogenetic activation (CHR2, bottom). Data from (*52*).
(B) Normalized expression of up-regulated genes upon two types of neuronal stimulation: KCl-induced depolarization (left) or optogenetically with CHR2 (right).
(C) Differentially expressed genes (DEG) between eMIC- and control adult brains. FC: fold-change, FDR: false discovery rate (adjusted p-values). In blue, genes regulated by sustained neuronal depolarization as identified in panel B.

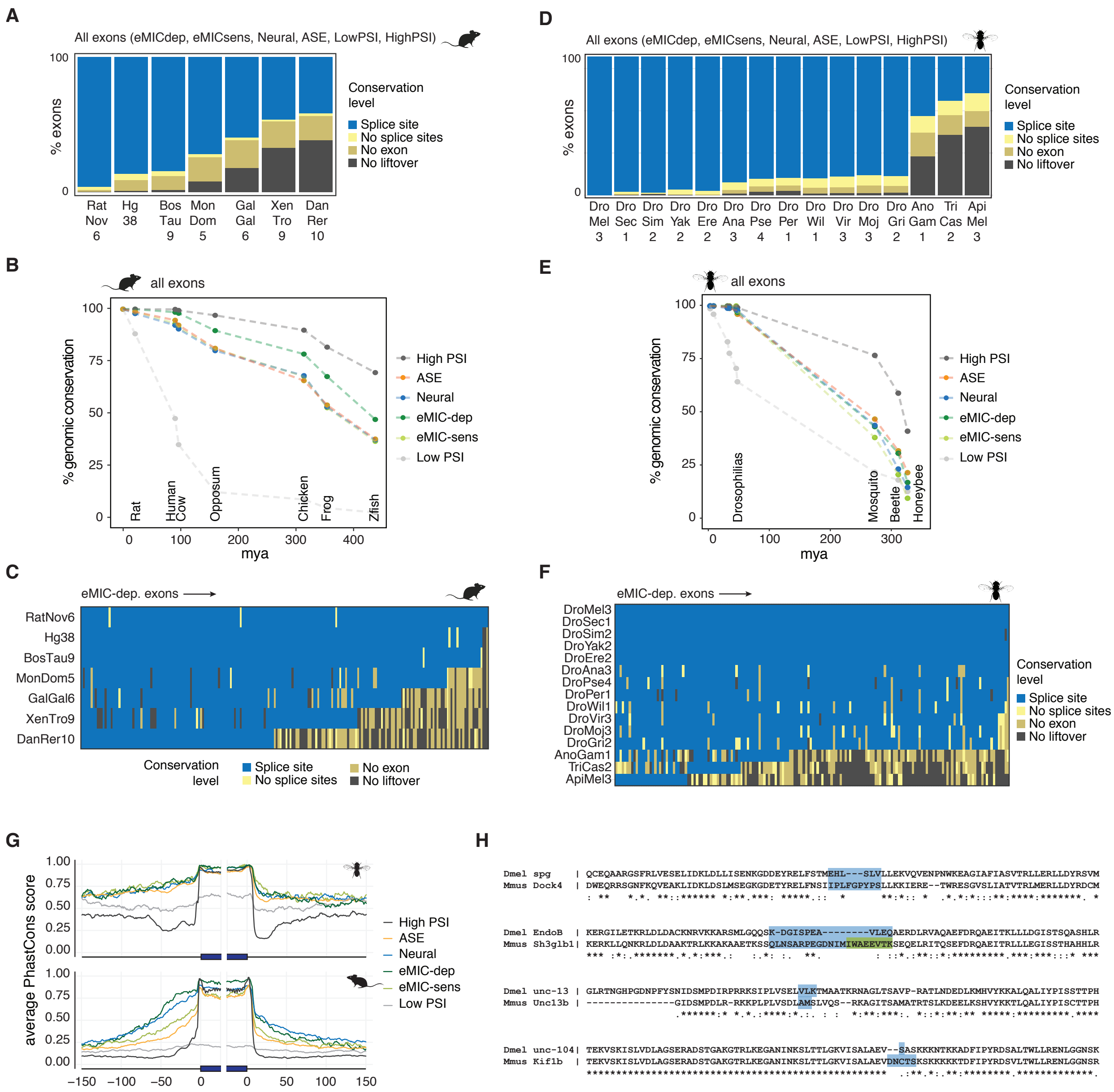

Supplementary Figure 11. Genomic conservation of eMIC-dependent exons

(A) Percentage of mouse exons conserved in the genome assemblies of other species as identified by liftOver.

(B) Genomic conservation of different types of exons. Y-axis represents percentage of exons with identified spliced sites between the focal species (mouse) and each other species, distributed on the x-axis according to the distance to their last common ancestor with the focal species. Related to Figure 9A, but without requiring liftOver of the adjacent constitutive exons. HighPSI: constitutive exons, ASE: alternatively spliced exons, Neural: neural-enriched exons, eMIC-dep and eMIC-sens: eMIC-dependent and sensitive exons, LowPSI: cryptic exons, mya: million years ago.

(C) Genomic conservation of each mouse eMIC-dependent exon in other vertebrate species.

(D-F) Equivalent analyses to panels A-C, done using *D. melanogaster* exons (dm6 assembly).

(G) Sequence conservation of the exonic and flanking intronic regions for six exon groups in mouse and *Drosophila* (Table S2 and Methods).
(H) Protein alignment in the region surrounding the exons regulated by the eMIC domain (highlighted with coloured boxes) for the only 4 exons shared between the fly and mammalian programs. Dmel: *Drosophila melanogaster*, Mmus: *Mus musculus*.

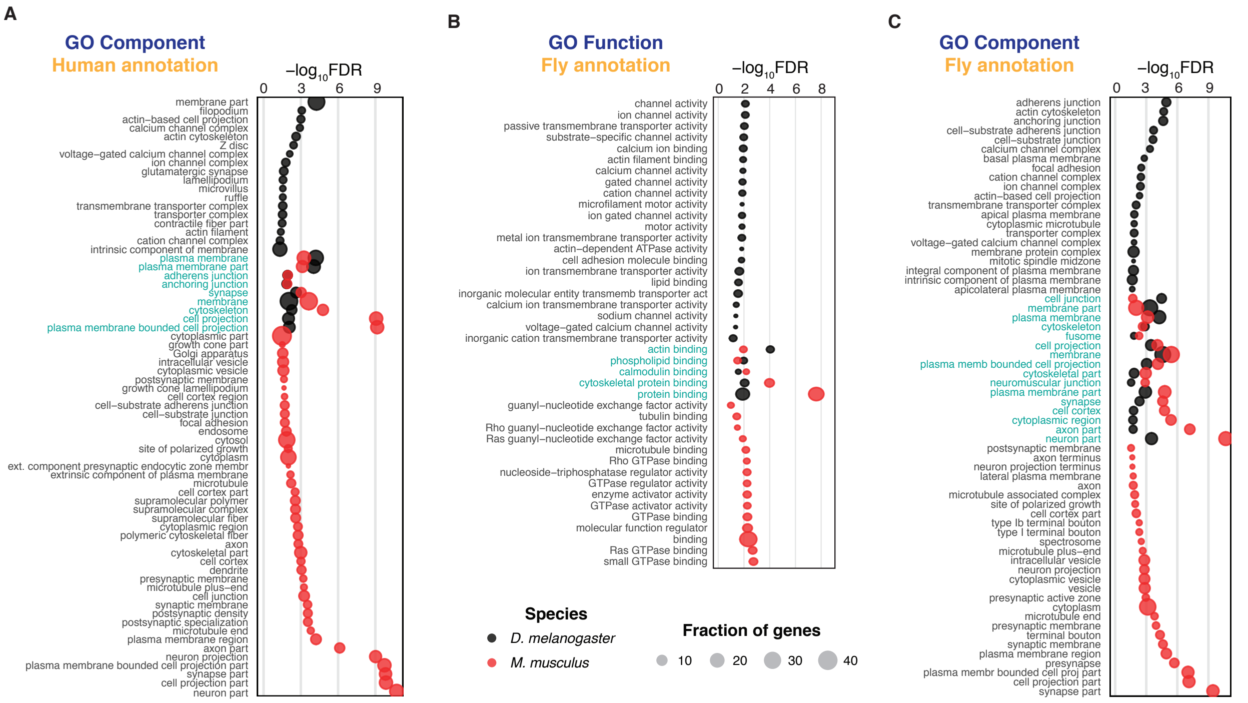

Supplementary Figure 12. Gene ontology (GO) terms enriched in eMIC targets

(A) GO terms in the Component categories for the human orthologs of *Drosophila* and mouse genes harbouring eMIC-dependent exons.

(B,C) Enrichment of GO terms for genes with eMIC exons, using the *Drosophila* GO annotation using either *Drosophila* genes or the *Drosophila* orthologs of mouse genes, for the Function (B) and Component (C) categories. FDR: False Discovery Rate, Fraction: percentage of genes with eMIC exons associated with each GO term. In blue, GO terms enriched in both *Drosophila* and mouse eMIC target genes.

Table S1. (separate file)

List of RNA-seq samples from *Drosophila*, human and mouse, analysed in this study.

Table S2. (separate file)

List of eMIC-dependent exons in *Drosophila* and mouse, and classification of the fly exons according to their profile across neural cell types.

Table S3. (separate file)

Gene Ontology (GO) terms enriched in *Drosophila* and mouse eMIC-dependent exons using the human GO annotation; and signalling- or gene regulation-related genes with eMIC-dependent exons in *Drosophila*.

Table S4. (separate file)

Differentially expressed genes in *Drosophila* eMIC- adult brain.

Table S5. (separate file)

List of primers and fly stocks used in this study.

Movie S1. (separate file)

Self-righting defects in eMIC- male adult flies.

Movie S2. (separate file)

Body posture and locomotion defects in *elav>Srrm234-I*;eMIC- male adult flies.

Movie S3. (separate file)

Wave amplitude of control and eMIC- larval central nervous system during fictive locomotion experiments.

REFERENCES AND NOTES

1. D. Schmucker, J. C. Clemens, H. Shu, C. A. Worby, J. Xiao, M. Muda, J. E. Dixon, S. L. Zipursky, Drosophila Dscam is an axon guidance receptor exhibiting extraordinary molecular diversity. *Cell* **101**, 671–684 (2000).

2. W. Gilbert, Why genes in pieces? *Nature* **271**, 501–501(1978).

3. S. J. Bush, L. Chen, J. M. Tovar-Corona, A. O. Urrutia, Alternative splicing and the evolution of phenotypic novelty. *Philos. Trans. R. Soc. Lond. B Biol. Sci.* **372**, 20150474 (2017).

4. N. L. Barbosa-Morais, M. Irimia, Q. Pan, H. Y. Xiong, S. Gueroussov, L. J. Lee, V. Slobodeniuc, C. Kutter, S. Watt, R. Çolak, T. H. Kim, C. M. Misquitta-Ali, M. D. Wilson, P. M. Kim, D. T. Odom, B. J. Frey, B. J. Blencowe, The evolutionary landscape of alternative splicing in vertebrate species. *Science* **338**, 1587–1593 (2012).

5. J. B. Brown, N. Boley, R. Eisman, G. E. May, M. H. Stoiber, M. O. Duff, B. W. Booth, J. Wen, S. Park, A. M. Suzuki, K. H. Wan, C. Yu, D. Zhang, J. W. Carlson, L. Cherbas, B. D. Eads, D. Miller, K. Mockaitis, J. Roberts, C. A. Davis, E. Frise, A. S. Hammonds, S. Olson, S. Shenker, D. Sturgill, A. A. Samsonova, R. Weiszmann, G. Robinson, J. Hernandez, J. Andrews, P. J. Bickel, P. Carninci, P. Cherbas, T. R. Gingeras, R. A. Hoskins, T. C. Kaufman, E. C. Lai, B. Oliver, N. Perrimon, B. R. Graveley, S. E. Celniker, Diversity and dynamics of the *Drosophila* transcriptome. *Nature* **512**, 393–399 (2014).

6. C. K. Vuong, D. L. Black, S. Zheng, The neurogenetics of alternative splicing. *Nat. Rev. Neurosci.* **17**, 265–281 (2016).

7. E. Furlanis, P. Scheiffele, Regulation of neuronal differentiation, function, and plasticity by alternative splicing. *Annu. Rev. Cell Dev. Biol.* **34**, 451–469 (2018).

8. B. Raj, B. J. Blencowe, Alternative splicing in the mammalian nervous system: Recent insights into mechanisms and functional roles. *Neuron* **87**, 14–27 (2015).

9. D. D. Licatalosi, R. B. Darnell, Splicing regulation in neurologic disease. *Neuron* **52**, 93–101 (2006).

10. S. Nik, T. V. Bowman, Splicing and neurodegeneration: Insights and mechanisms. *Wiley Interdiscip. Rev. RNA* **10**, e1532 (2019).

11. M. Irimia, R. J. Weatheritt, J. D. Ellis, N. N. Parikshak, T. Gonatopoulos-Pournatzis, M. Babor, M. Quesnel-Vallières, J. Tapial, B. Raj, D. O'Hanlon, M. Barrios-Rodiles, M. J. E. Sternberg, S. P. Cordes, F. P. Roth, J. L. Wrana, D. H. Geschwind, B. J. Blencowe, A highly conserved program of neuronal microexons is misregulated in autistic brains. *Cell* **159**, 1511–1523 (2014).

12. M. Quesnel-Vallières, M. Irimia, S. P. Cordes, B. J. Blencowe, Essential roles for the splicing regulator nSR100/SRRM4 during nervous system development. *Genes Dev.* **29**, 746–759 (2015).

13. M. Quesnel-Vallières, Z. Dargaei, M. Irimia, T. Gonatopoulos-Pournatzis, J. Y. Ip, M. Wu, T. Sterne-Weiler, S. Nakagawa, M. A. Woodin, B. J. Blencowe, S. P. Cordes, Misregulation of an activity-dependent splicing network as a common mechanism underlying autism spectrum disorders. *Mol. Cell* **64**, 1023–1034 (2016).

14. Y. Nakano, S. Wiechert, B. Bánfi, Overlapping activities of two neuronal splicing factors switch the GABA effect from excitatory to inhibitory by regulating REST. *Cell Rep.* **27**, 860–871.e8 (2019).

15. A. Torres-Méndez, S. Bonnal, Y. Marquez, J. Roth, M. Iglesias, J. Permanyer, I. Almudí, D. O'Hanlon, T. Guitart, M. Soller, A. C. Gingras, F. Gebauer, F. Rentzsch, B. J. Blencowe, J. Valcárcel, M. Irimia, A novel protein domain in an ancestral splicing factor drove the evolution of neural microexons. *Nat. Ecol. Evol.* **3**, 691–701 (2019).

16. Y. I. Li, L. Sanchez-Pulido, W. Haerty, C. P. Ponting, RBFOX and PTBP1 proteins regulate the alternative splicing of micro-exons in human brain transcripts. *Genome Res.* **25**, 1–13 (2015).

17. B. Raj, M. Irimia, U. Braunschweig, T. Sterne-Weiler, D. O'Hanlon, Z. Y. Lin, G. I. Chen, L. E. Easton, J. Ule, A. C. Gingras, E. Eyras, B. J. Blencowe, A global regulatory mechanism for activating an exon network required for neurogenesis. *Mol. Cell* **56**, 90–103 (2014).

18. T. Gonatopoulos-Pournatzis, M. Wu, U. Braunschweig, J. Roth, H. Han, A. J. Best, B. Raj, M. Aregger, D. O'Hanlon, J. D. Ellis, J. A. Calarco, J. Moffat, A. C. Gingras, B. J. Blencowe, Genome-

wide CRISPR-Cas9 interrogation of splicing networks reveals a mechanism for recognition of autism-misregulated neuronal microexons. *Mol. Cell* **72**, 510–524.e12 (2018).

19. D. P. Leader, S. A. Krause, A. Pandit, S. A. Davies, J. A. T. Dow, FlyAtlas 2: A new version of the *Drosophila melanogaster* expression atlas with RNA-Seq, miRNA-Seq and sex-specific data. *Nucleic Acids Res.* **46**, D809–D815 (2018).

20. R. Barrio, J. F. De Celis, Regulation of spalt expression in the Drosophila wing blade in response to the Decapentaplegic signaling pathway. *Proc. Natl. Acad. Sci. U.S.A.* **101**, 6021–6026 (2004).

21. T. Ikeya, M. Galic, P. Belawat, K. Nairz, E. Hafen, Nutrient-dependent expression of insulin-like peptides from neuroendocrine cells in the CNS contributes to growth regulation in Drosophila. *Curr. Biol.* **12**, 1293–1300 (2002).

22. J. Lee, C. F. Wu, Electroconvulsive seizure behavior in Drosophila: Analysis of the physiological repertoire underlying a stereotyped action pattern in bang-sensitive mutants. *J. Neurosci.* **22**, 11065–11079 (2002).

23. H. Lacin, H. M. Chen, X. Long, R. H. Singer, T. Lee, J. W. Truman, Neurotransmitter identity is acquired in a lineage-restricted manner in the *Drosophila* CNS. *eLife* **8**, e43701 (2019).

24. A. Mahr, H. Aberle, The expression pattern of the Drosophila vesicular glutamate transporter: A marker protein for motoneurons and glutamatergic centers in the brain. *Gene Expr. Patterns* **6**, 299–309 (2006).

25. S. R. Pulver, T. G. Bayley, A. L. Taylor, J. Berni, M. Bate, B. Hedwig, Imaging fictive locomotor patterns in larval Drosophila. *J. Neurophysiol.* **114**, 2564–2577 (2015).

26. W. C. Lemon, S. R. Pulver, B. Höckendorf, K. McDole, K. Branson, J. Freeman, P. J. Keller, Whole-central nervous system functional imaging in larval *Drosophila*. *Nat. Commun.* **6**, 7924 (2015).

27. S. Y. Kao, E. Nikonova, K. Ravichandran, M. L. Spletter, Dissection of *Drosophila melanogaster* flight muscles for omics approaches. *J. Vis. Exp.* **2019**, e60309 (2019).

28. M. S. Shiao, J. M. Chang, W. L. Fan, M. Y. J. Lu, C. Notredame, S. Fang, R. Kondo, W. H. Li, Expression divergence of chemosensory genes between *Drosophila sechellia* and its sibling species and its implications for host shift. *Genome Biol. Evol.* **7**, 2843–2858 (2015).

29. L. Gibilisco, Q. Zhou, S. Mahajan, D. Bachtrog, Alternative splicing within and between Drosophila species, sexes, tissues, and developmental stages. *PLOS Genet.* **12**, e1006464 (2016).

30. J. Tapial, K. C. H. Ha, T. Sterne-Weiler, A. Gohr, U. Braunschweig, A. Hermoso-Pulido, M. Quesnel-Vallières, J. Permanyer, R. Sodaei, Y. Marquez, L. Cozzuto, X. Wang, M. Gómez-Velázquez, T. Rayon, M. Manzanares, J. Ponomarenko, B. J. Blencowe, M. Irimia, An atlas of alternative splicing profiles and functional associations reveals new regulatory programs and genes that simultaneously express multiple major isoforms. *Genome Res.* **27**, 1759–1768 (2017).

31. L. De Conti, M. Baralle, E. Buratti, Exon and intron definition in pre-mRNA splicing. *Wiley Interdiscip. Rev. RNA* **4**, 49–60(2013).

32. A. A. Pai, T. Henriques, K. McCue, A. Burkholder, K. Adelman, C. B. Burge, The kinetics of pre-mRNA splicing in the Drosophila genome and the influence of gene architecture. *eLife* **6**, e32537 (2017).

33. J. Heimiller, V. Sridharan, J. Huntley, C. S. Wesley, R. Singh, Drosophila polypyrimidine tract-binding protein (DmPTB) regulates dorso-ventral patterning genes in embryos. *PLOS ONE* **9**, e98585 (2014).

34. A. N. Brooks, M. O. Duff, G. May, L. Yang, M. Bolisetty, J. Landolin, K. Wan, J. Sandler, B. W. Booth, S. E. Celniker, B. R. Graveley, S. E. Brenner, Regulation of alternative splicing in Drosophila by 56 RNA binding proteins. *Genome Res.* **25**, 1771–1780 (2015).

35. A. N. Brooks, L. Yang, M. O. Duff, K. D. Hansen, J. W. Park, S. Dudoit, S. E. Brenner, B. R. Graveley, Conservation of an RNA regulatory map between Drosophila and mammals. *Genome Res.* **21**, 193–202 (2011).

36. M. O. Duff, S. Olson, X. Wei, S. C. Garrett, A. Osman, M. Bolisetty, A. Plocik, S. E. Celniker, B. R. Graveley, Genome-wide identification of zero nucleotide recursive splicing in Drosophila. *Nature* **521**, 376–379 (2015).

37. J. Kralovicova, I. Vorechovsky, Alternative splicing of U2AF1 reveals a shared repression mechanism for duplicated exons. *Nucleic Acids Res.* **45**, 417–434 (2017).

38. J. Ule, B. J. Blencowe, Alternative splicing regulatory networks: Functions, mechanisms, and evolution. *Mol. Cell* **76**, 329–345 (2019).

39. M. H. Stoiber, S. Olson, G. E. May, M. O. Duff, J. Manent, R. Obar, K. G. Guruharsha, P. J. Bickel, S. Artavanis-Tsakonas, J. B. Brown, B. R. Graveley, S. E. Celniker, Extensive cross-regulation of post-transcriptional regulatory networks in Drosophila. *Genome Res.* **25**, 1692–1702 (2015).

40. A. L. Sapiro, E. C. Freund, L. Restrepo, H. H. Qiao, A. Bhate, Q. Li, J. Q. Ni, T. J. Mosca, J. B. Li, Zinc finger RNA-binding protein Zn72D regulates ADAR-mediated RNA editing in neurons. *Cell Rep.* **31**, 107654 (2020).

41. L. Wei, S. Lee, S. Majumdar, B. Zhang, P. Sanfilippo, B. Joseph, P. Miura, M. Soller, E. C. Lai, Overlapping activities of ELAV/Hu family RNA binding proteins specify the extended neuronal 3′ UTR landscape in Drosophila. *Mol. Cell* **80**, 140–155.e6 (2020).

42. J. Carrasco, M. Rauer, B. Hummel, D. Grzejda, C. Alfonso-Gonzalez, Y. Lee, Q. Wang, M. Puchalska, G. Mittler, V. Hilgers, ELAV and FNE determine neuronal transcript signatures through EXon-activated rescue. *Mol. Cell* **80**, 156–163.e6 (2020).

43. V. Hilgers, S. B. Lemke, M. Levine, ELAV mediates 3′ UTR extension in the Drosophila nervous system. *Genes Dev.* **26**, 2259–2264 (2012).

44. S. P. Koushika, M. Soller, K. White, The neuron-enriched splicing pattern of Drosophila erect wing is dependent on the presence of ELAV protein. *Mol. Cell. Biol.* **20**, 1836–1845 (2000).

45. R. S. Porter, F. Jaamour, S. Iwase, Neuron-specific alternative splicing of transcriptional machineries: Implications for neurodevelopmental disorders. *Mol. Cell. Neurosci.* **87**, 35–45 (2018).

46. B. T. Cocanougher, J. D. Wittenbach, X. S. Long, A. B. Kohn, T. P. Norekian, J. Yan, J. Colonell, J. B. Masson, J. W. Truman, A. Cardona, S. C. Turaga, R. H. Singer, L. L. Moroz, M. Zlatic, Comparative single-cell transcriptomics of complete insect nervous systems. *bioRxiv* 785931 (2020).

47. C. B. Avalos, R. Brugmann, S. G. Sprecher, Single cell transcriptome atlas of the *Drosophila* larval brain. *eLife* **8**, e50354 (2019).

48. F. P. Davis, A. Nern, S. Picard, M. B. Reiser, G. M. Rubin, S. R. Eddy, G. L. Henry, A genetic, genomic, and computational resource for exploring neural circuit function. *eLife* **9**, e50901 (2020).

49. G. L. Henry, F. P. Davis, S. Picard, S. R. Eddy, Cell type-specific genomics of Drosophila neurons. *Nucleic Acids Res.* **40**, 9691–9704 (2012).

50. M. F. M. Shih, F. P. Davis, G. L. Henry, J. Dubnau, Nuclear transcriptomes of the seven neuronal cell types that constitute the Drosophila mushroom bodies. *G3* **9**, 81–94 (2019).

51. H. Hall, J. Ma, S. Shekhar, W. D. Leon-Salas, V. M. Weake, Blue light induces a neuroprotective gene expression program in *Drosophila* photoreceptors. *BMC Neurosci.* **19**, 43 (2018).

52. X. Chen, R. Rahman, F. Guo, M. Rosbash, Genome-wide identification of neuronal activity-regulated genes in Drosophila. *eLife* **5**, e19942 (2016).

53. M. Irimia, J. L. Rukov, S. W. Roy, J. Vinther, J. Garcia-Fernandez, Quantitative regulation of alternative splicing in evolution and development. *Bioessays* **31**, 40–50 (2009).

54. B. R. Graveley, A. N. Brooks, J. W. Carlson, M. O. Duff, J. M. Landolin, L. Yang, C. G. Artieri, M. J. Van Baren, N. Boley, B. W. Booth, J. B. Brown, L. Cherbas, C. A. Davis, A. Dobin, R. Li, W. Lin, J. H. Malone, N. R. Mattiuzzo, D. Miller, D. Sturgill, B. B. Tuch, C. Zaleski, D. Zhang, M. Blanchette, S. Dudoit, B. Eads, R. E. Green, A. Hammonds, L. Jiang, P. Kapranov, L. Langton, N. Perrimon, J. E. Sandler, K. H. Wan, A. Willingham, Y. Zhang, Y. Zou, J. Andrews, P. J. Bickel, S. E. Brenner, M. R. Brent, P. Cherbas, T. R. Gingeras, R. A. Hoskins, T. C. Kaufman, B. Oliver, S. E. Celniker, The developmental transcriptome of *Drosophila melanogaster*. *Nature* **471**, 473–479 (2011).

55. I. Kelava, F. Rentzsch, U. Technau, Evolution of eumetazoan nervous systems: Insights from cnidarians. *Philos. Trans. R. Soc. Lond. Biol. Sci.* **370**, 20150065 (2015).

56. C. Colombrita, V. Silani, A. Ratti, ELAV proteins along evolution: Back to the nucleus? *Mol. Cell. Neurosci.* **56**, 447–455 (2013).

57. E. Petruccelli, T. Brown, A. Waterman, N. Ledru, K. R. Kaun, Alcohol causes lasting differential transcription in *Drosophila* mushroom body neurons. *Genetics* **215**, 103–116 (2020).

58. Q. Wang, K. C. Abruzzi, M. Rosbash, D. C. Rio, Striking circadian neuron diversity and cycling of *Drosophila* alternative splicing. *eLife* **7**, e35618 (2018).

59. L. F. George, S. J. Pradhan, D. Mitchell, M. Josey, J. Casey, M. T. Belus, K. N. Fedder, G. Raj Dahal, E. A. Bates, Ion channel contributions to wing development in *Drosophila melanogaster*. *G3* **9**, 999–1008 (2019).

60. P. Ustaoglu, I. U. Haussmann, H. Liao, A. Torres-Mendez, R. Arnold, M. Irimia, M. Soller, Srrm234, but not canonical SR and hnRNP proteins, drive inclusion of *Dscam* exon 9 variable exons. *RNA* **25**, 1353–1365 (2019).

61. M. Irimia, A. Denuc, D. Burguer, I. Somorjai, J. M. Martín-Durán, G. Genikhovich, S. Jimenez-Delgado, U. Technau, S. W. Roy, G. Marfany, J. Garcia-Fernàndez, Stepwise assembly of the *Nova*-regulated alternative splicing network in the vertebrate brain. *Proc. Natl. Acad. Sci. U.S.A.* **108**, 5319–5324 (2011).

62. D. Burguera, Y. Marquez, C. Racioppi, J. Permanyer, A. Torres-Méndez, R. Esposito, B. Albuixech-Crespo, L. Fanlo, Y. D'Agostino, A. Gohr, E. Navas-Perez, A. Riesgo, C. Cuomo, G. Benvenuto, L. A. Christiaen, E. Martí, S. D'Aniello, A. Spagnuolo, F. Ristoratore, M. I. Arnone, J. Garcia-Fernàndez, M. Irimia, Evolutionary recruitment of flexible Esrp-dependent splicing programs into diverse embryonic morphogenetic processes. *Nat. Commun.* **8**, (2017).

63. Y. Márquez, F. Mantica, L. Cozzuto, D. Burguera, A. Hermoso-Pulido, J. Ponomarenko, S. W. Roy, M. Irimia, ExOrthist: A tool to infer exon orthologies at any evolutionary distance. *Genome Biol.* **22**, 239 (2021).

64. M. A. Yoshida, K. Yura, A. Ogura, Cephalopod eye evolution was modulated by the acquisition of Pax-6 splicing variants. *Sci. Rep.* **4**, 4256 (2014).

65. J. Wexler, E. K. Delaney, X. Belles, C. Schal, A. Wada-Katsumata, M. J. Amicucci, A. Kopp, Hemimetabolous insects elucidate the origin of sexual development via alternative splicing. *eLife* **8**, e47490 (2019).

66. H. Sakamoto, K. Inoue, I. Higuchi, Y. Ono, Y. Shimura, Control of Drosophila sex-lethal pre-mRNA splicing by its own female-specific product. *Nucleic Acids Res.* **20**, 5533–5540 (1992).

67. D. J. McLean, M. A. Skowron Volpon, trajr: An R package for characterisation of animal trajectories. *Ethology* **124**, 440–448 (2018).

68. J. Schindelin, I. Arganda-Carreras, E. Frise, V. Kaynig, M. Longair, T. Pietzsch, S. Preibisch, C. Rueden, S. Saalfeld, B. Schmid, J. Y. Tinevez, D. J. White, V. Hartenstein, K. Eliceiri, P. Tomancak, A. Cardona, Fiji: An open-source platform for biological-image analysis. *Nat. Methods* **9**, 676–682 (2012).

69. L. Y. Jan, Y. N. Jan, Antibodies to horseradish peroxidase as specific neuronal markers in Drosophila and in grasshopper embryos. *Proc. Natl. Acad. Sci. U.S.A.* **79**, 2700–2704 (1982).

70. C. Tischer, A. Ravindran, S. Reither, N. Chiaruttini, R. Pepperkok, N. Norlin, BigDataProcessor2: A free and open-source Fiji plugin for inspection and processing of TB sized image data. *Bioinformatics* **37**, 3079–3081 (2021).

71. P. Thévenaz, U. E. Ruttimann, M. Unser, A pyramid approach to subpixel registration based on intensity. *IEEE Trans. Image Process.* **7**, 27–41 (1998).

72. D. Garrido-Martín, E. Palumbo, R. Guigó, A. Breschi, ggsashimi: Sashimi plot revised for browser- and annotation-independent splicing visualization. *PLoS Comput. Biol.* **14**, e1006360 (2018).

73. S. A. Head, X. Hernandez-Alias, J.-S. S. Yang, L. Ciampi, V. Beltran-Sastre, A. Torres-Méndez, M. Irimia, M. H. Schaefer, L. Serrano, Silencing of SRRM4 suppresses microexon inclusion and promotes tumor growth across cancers. *PLOS Biol.* **19**, e3001138 (2021).

74. A. Gohr, M. Irimia, Matt: Unix tools for alternative splicing analysis. *Bioinformatics* **35**, 130–132 (2019).

75. S. Gueroussov, T. Gonatopoulos-Pournatzis, M. Irimia, B. Raj, Z. Y. Lin, A. C. Gingras, B. J. Blencowe, An alternative splicing event amplifies evolutionary differences between vertebrates. *Science* **349**, 868–873 (2015).

76. K. S. Hubbard, I. M. Gut, M. E. Lyman, P. M. McNutt, Longitudinal RNA sequencing of the deep transcriptome during neurogenesis of cortical glutamatergic neurons from murine ESCs. *F1000Res.* **2**, 35 (2013).

77. P. Sanfilippo, J. Wen, E. C. Lai, Landscape and evolution of tissue-specific alternative polyadenylation across Drosophila species. *Genome Biol.* **18**, 229 (2017).

78. A. J. Linares, C. H. Lin, A. Damianov, K. L. Adams, B. G. Novitch, D. L. Black, The splicing regulator PTBP1 controls the activity of the transcription factor Pbx1 during neuronal differentiation. *eLife* **4**, e09268 (2015).
